# Supplementary figures and images for: Immediate postnatal prediction of death or bronchopulmonary dysplasia among very preterm and very low birth weight infants based on gradient boosting decision trees algorithm: A nationwide database study in Japan
Source: PLoS One. 2024 Mar 27;19(3):e0300817. doi: 10.1371/journal.pone.0300817 (PMC10971761; doi:10.1371/journal.pone.0300817)

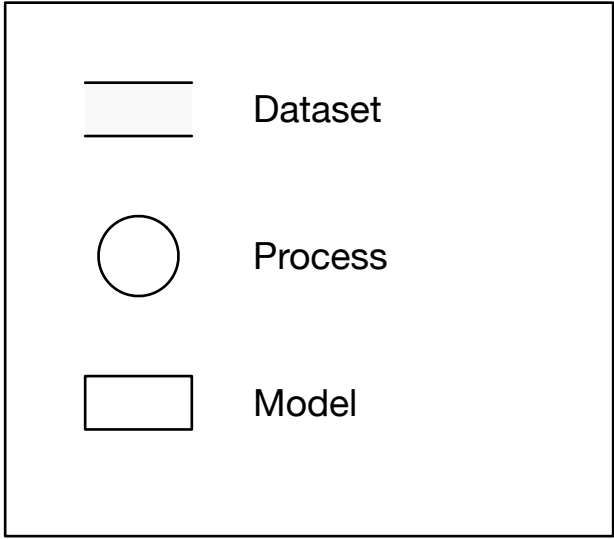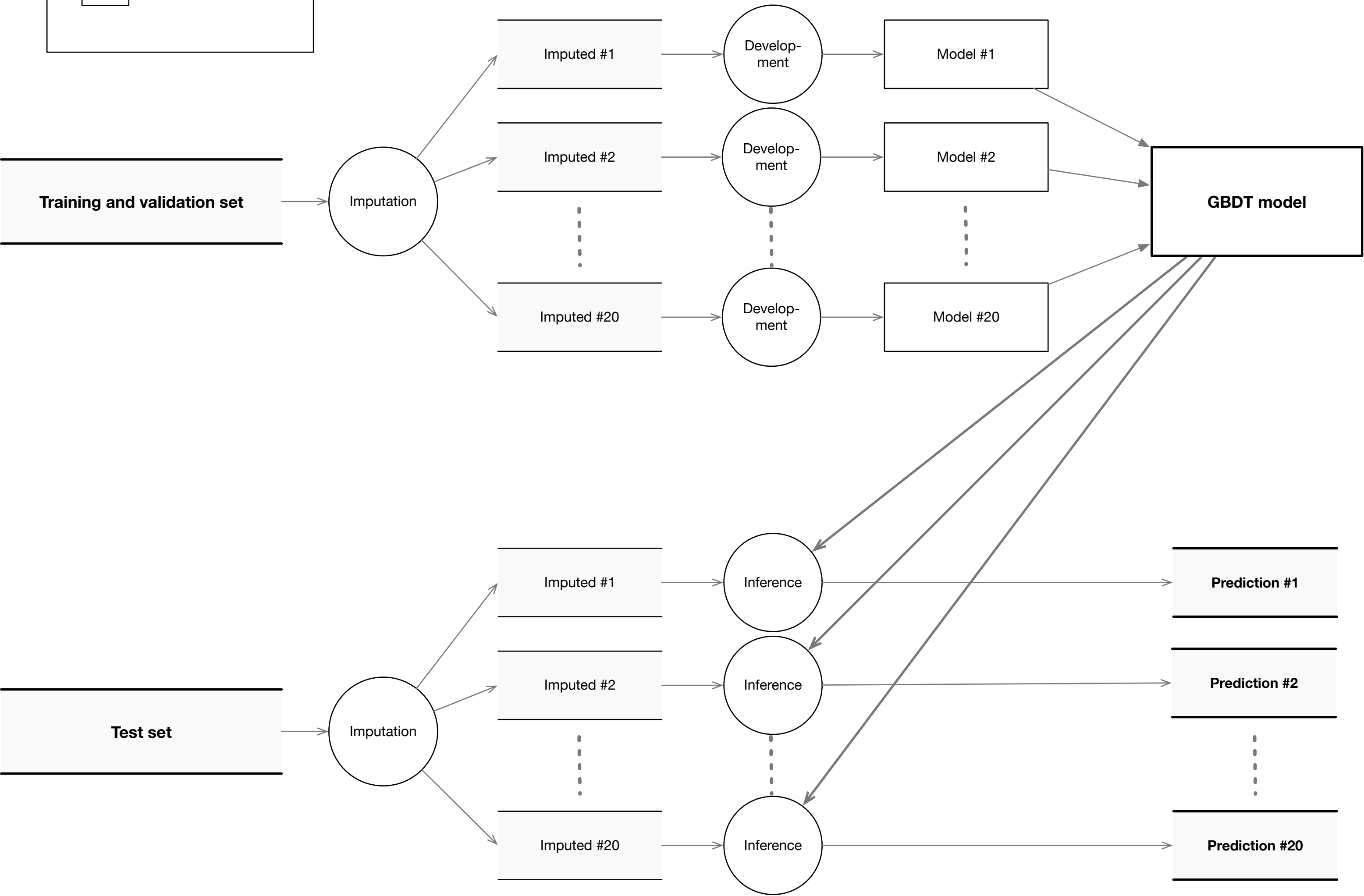

Supplement: S1 Fig — (PDF) [file pone.0300817.s001.pdf]

**GBDT Model**

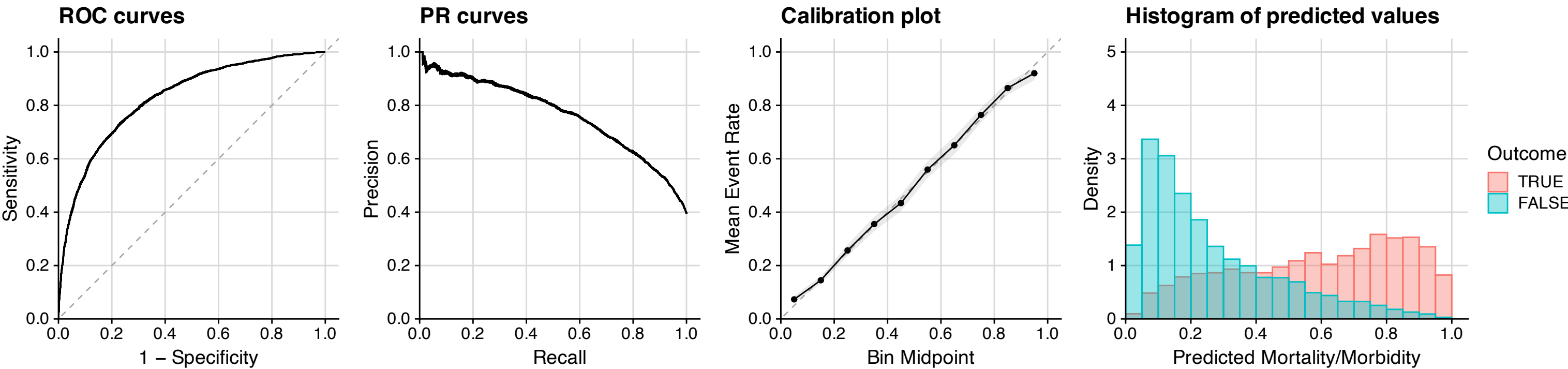

**Multivariable Logistic Regression Model**

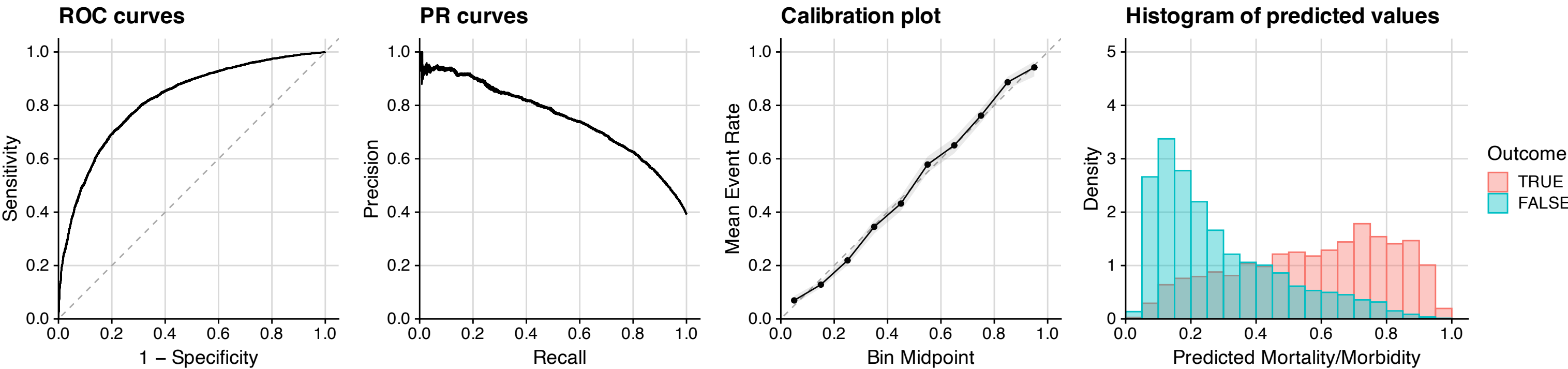

Supplement: S2 Fig — The upper panel shows the gradient boosting decision trees model, and the lower panel shows the multivariable logistic regression model. For each model, receiver operating characteristic curves, precision-recall curves, calibration plots, and histograms of predicted values are aligned from left to right. (PDF) [file pone.0300817.s002.pdf]

**GBDT Model**

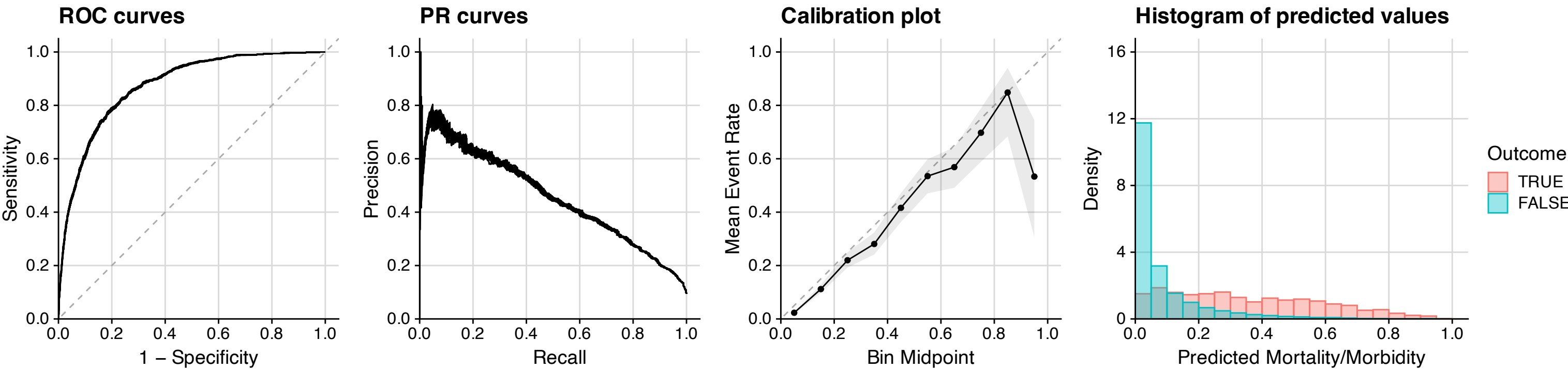

**Multivariable Logistic Regression Model**

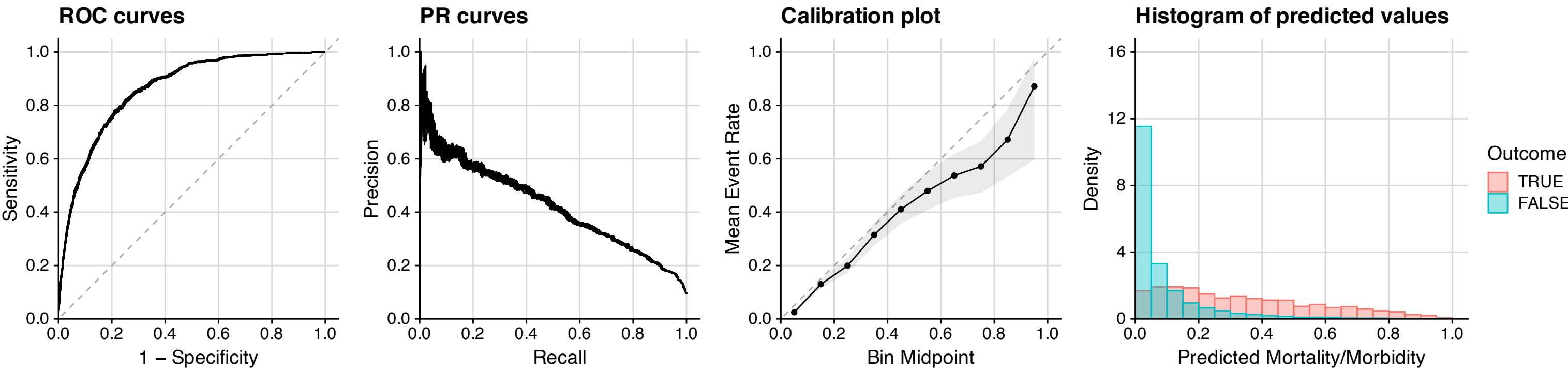

Supplement: S3 Fig — The upper panel shows the gradient boosting decision trees model, and the lower panel shows the multivariable logistic regression model. For each model, receiver operating characteristic curves, precision-recall curves, calibration plots, and histograms of predicted values are aligned from left to right. (PDF) [file pone.0300817.s003.pdf]

**GBDT Model**

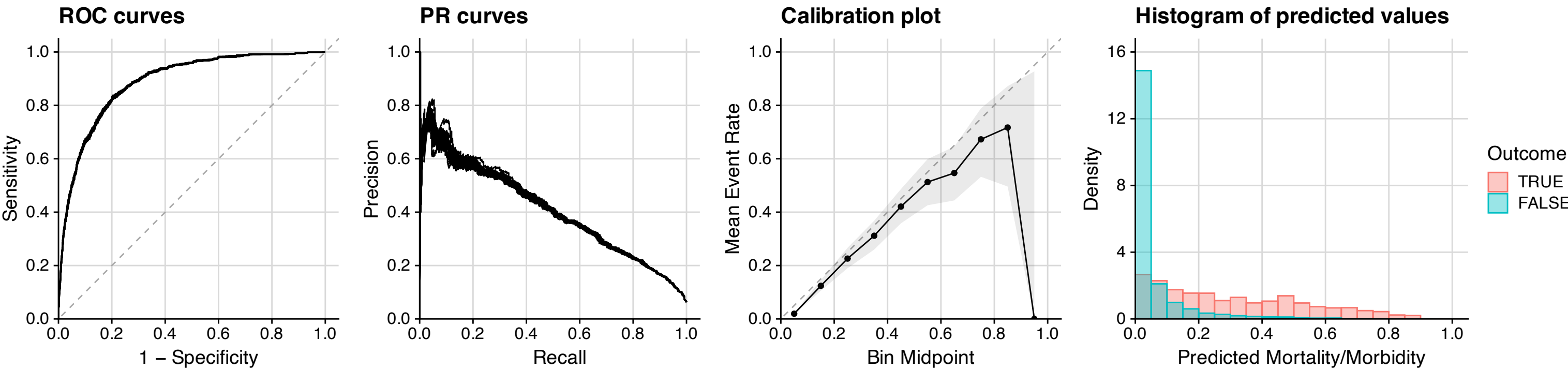

**Multivariable Logistic Regression Model**

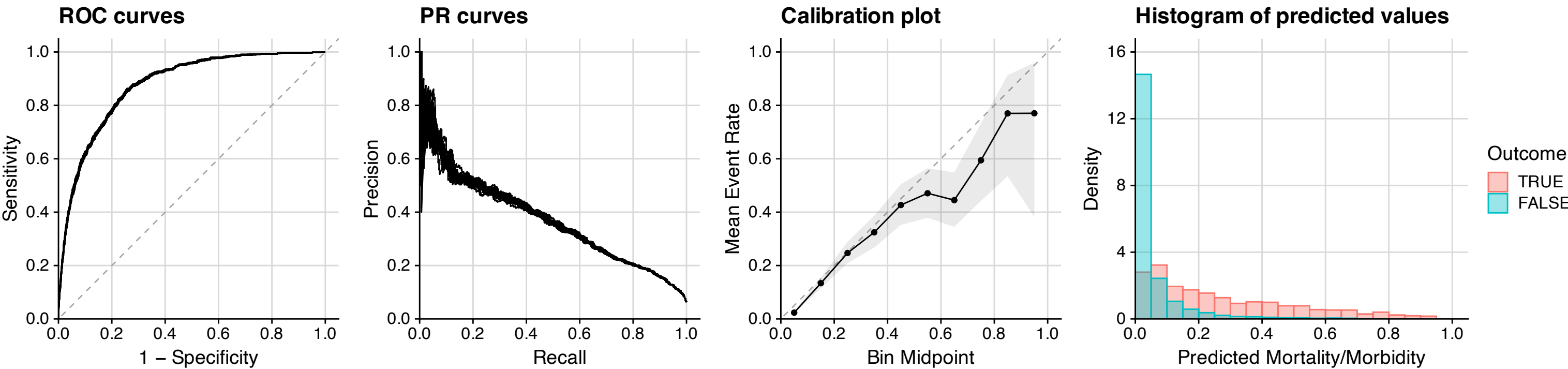

Supplement: S4 Fig — The upper panel shows the gradient boosting decision trees model, and the lower panel shows the multivariable logistic regression model. For each model, receiver operating characteristic curves, precision-recall curves, calibration plots, and histograms of predicted values are aligned from left to right. (PDF) [file pone.0300817.s004.pdf]

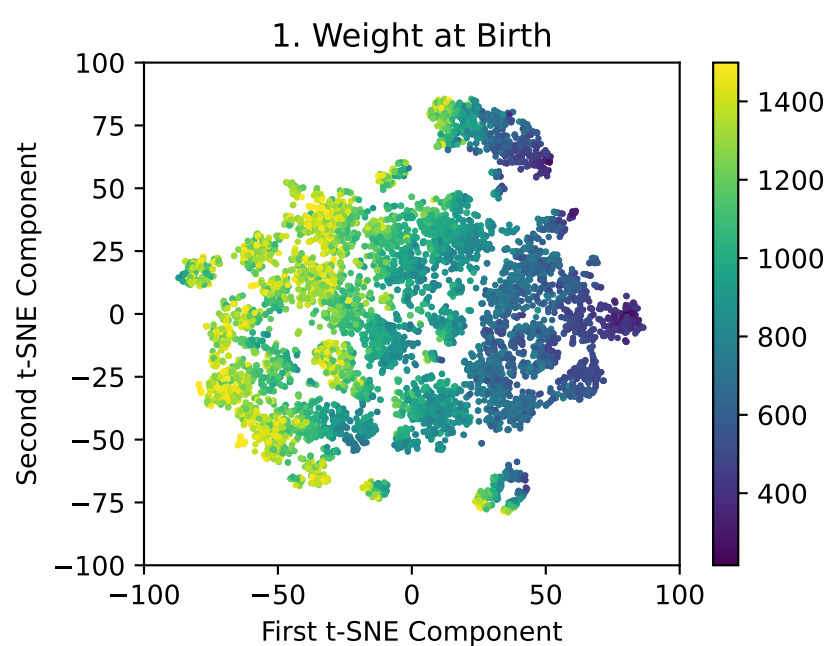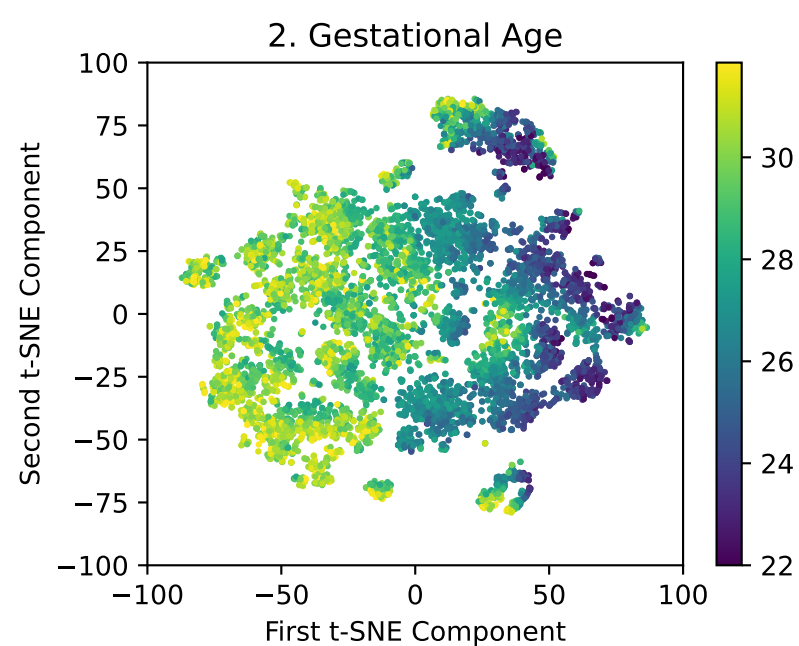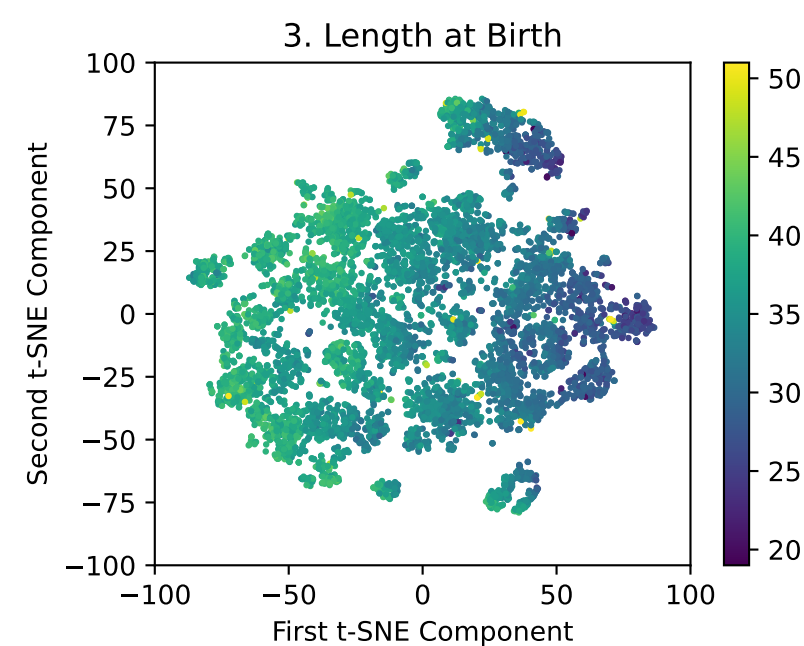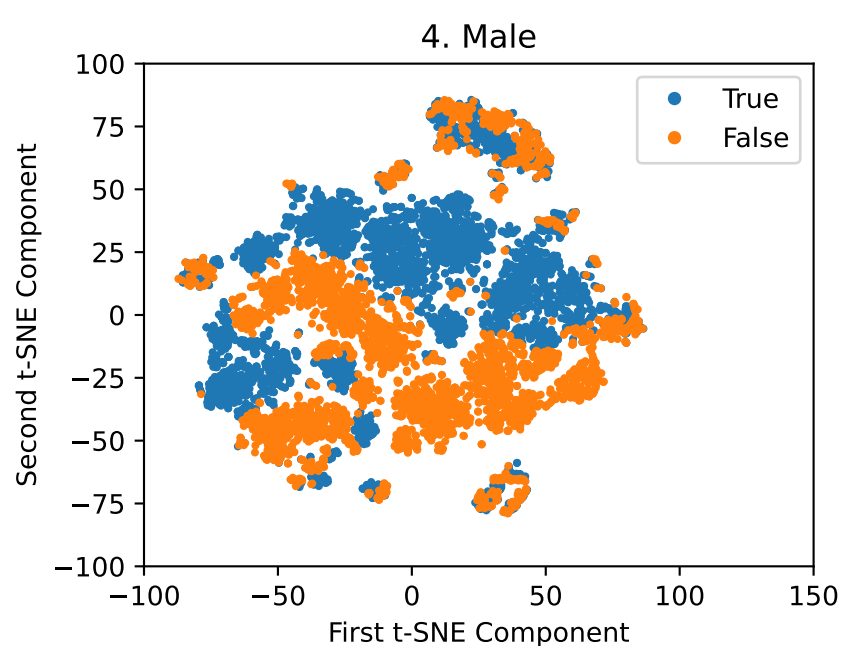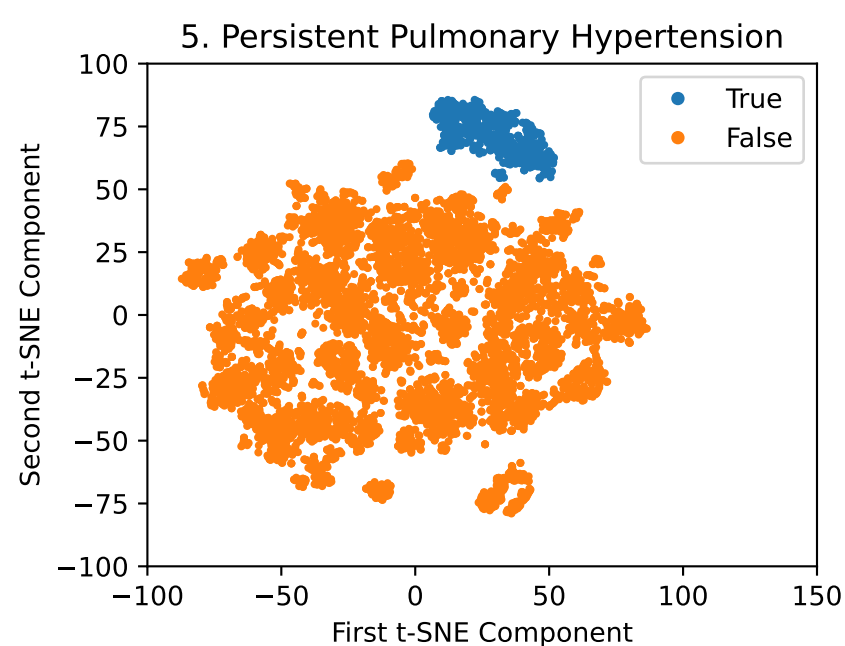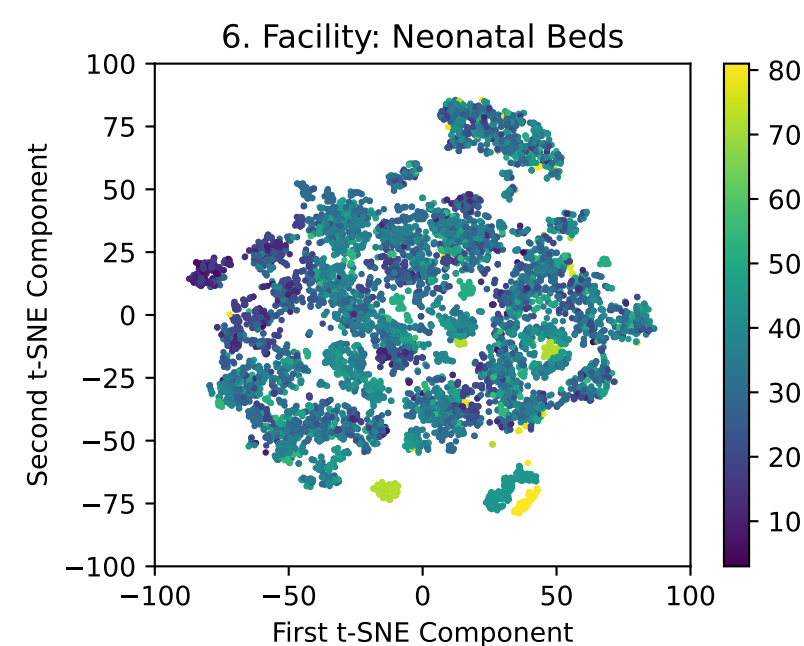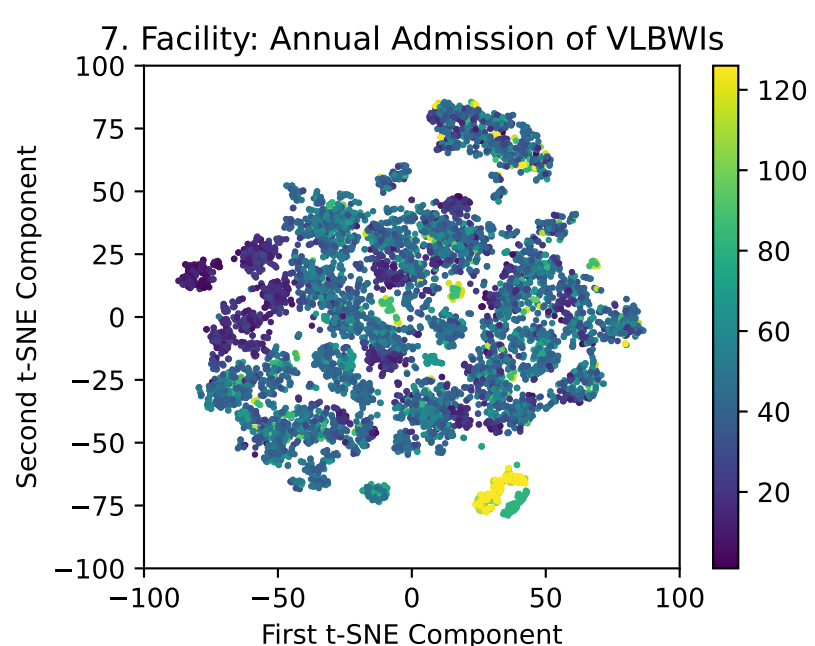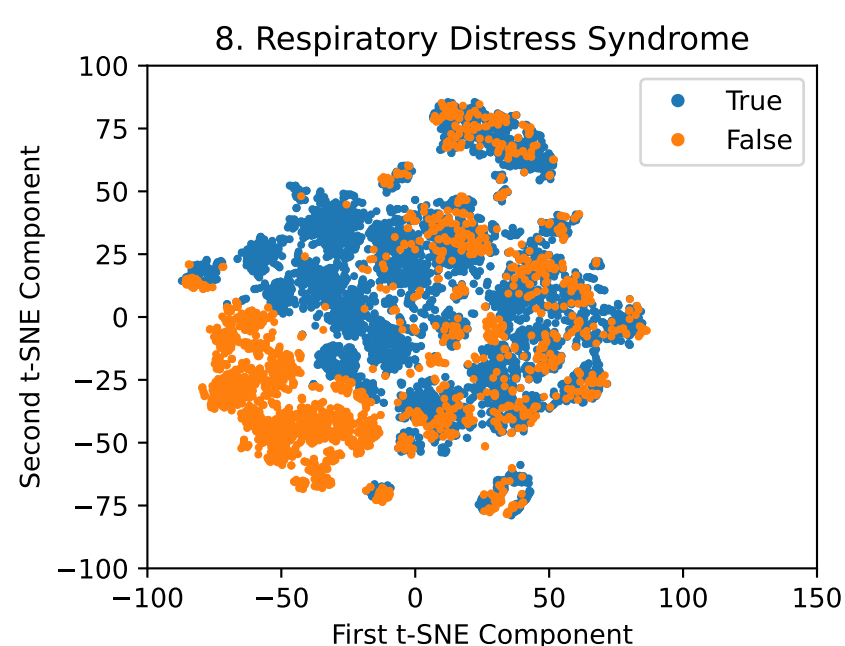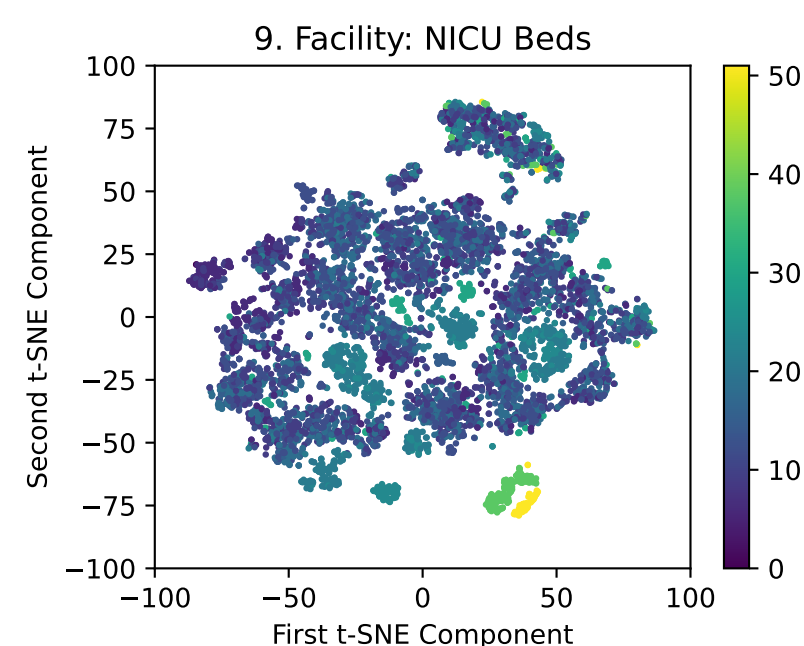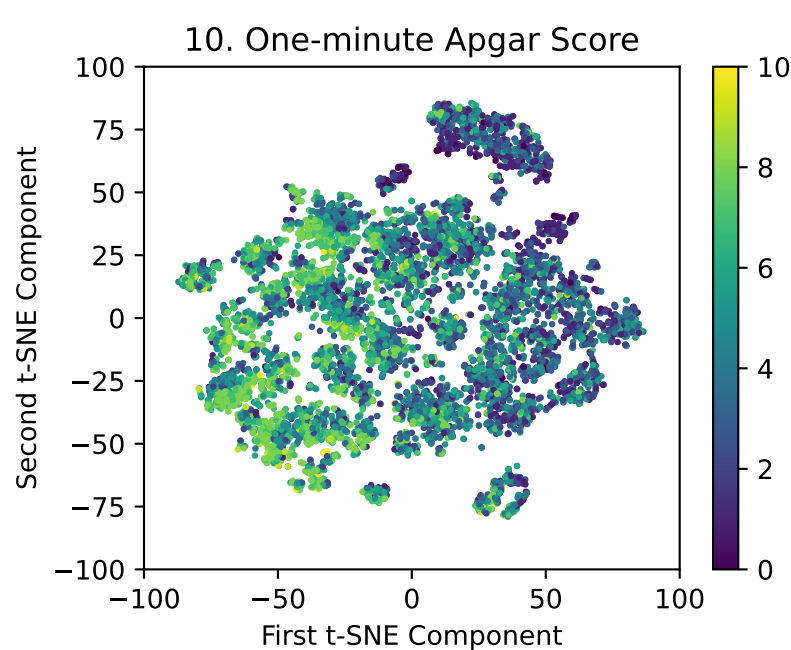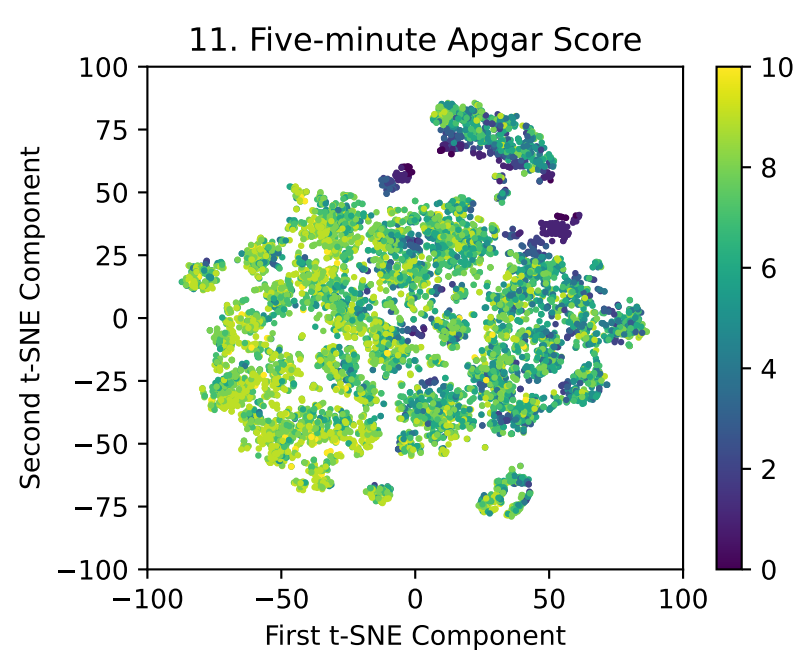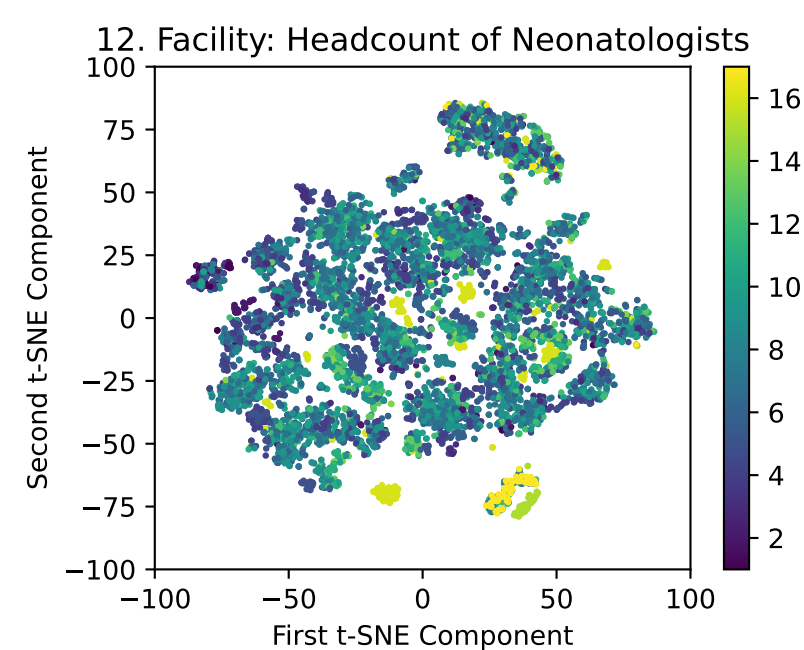

Supplement: S8 Fig — Derived from the SHAP values of the first imputed test set. (PDF) [file pone.0300817.s008.pdf]

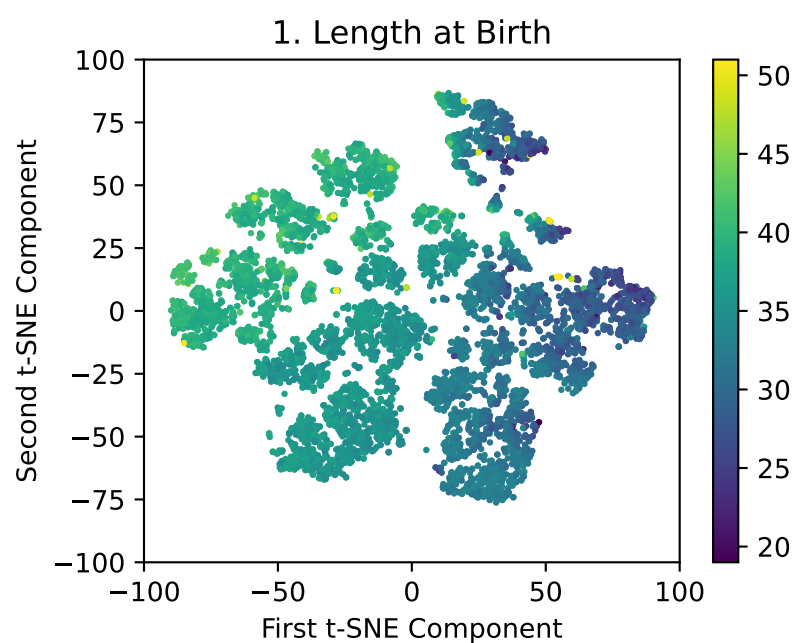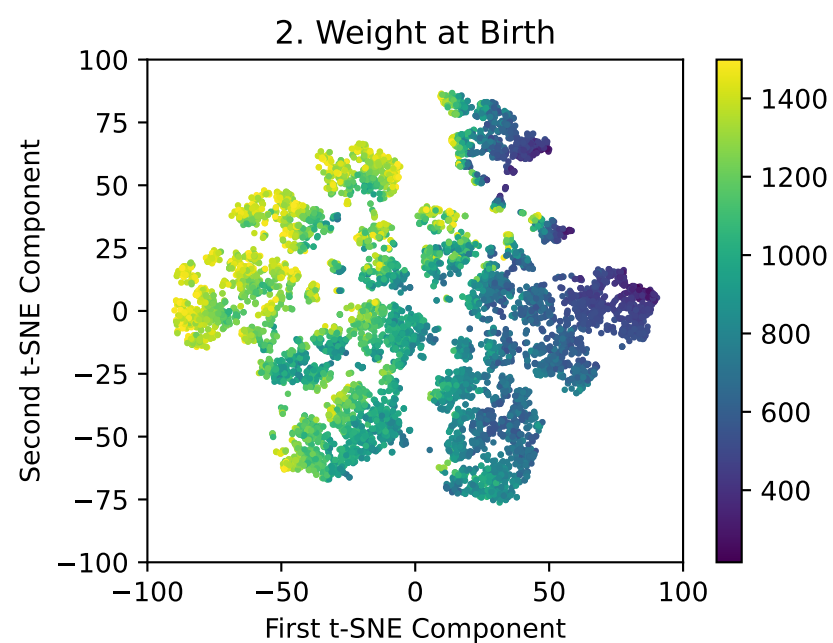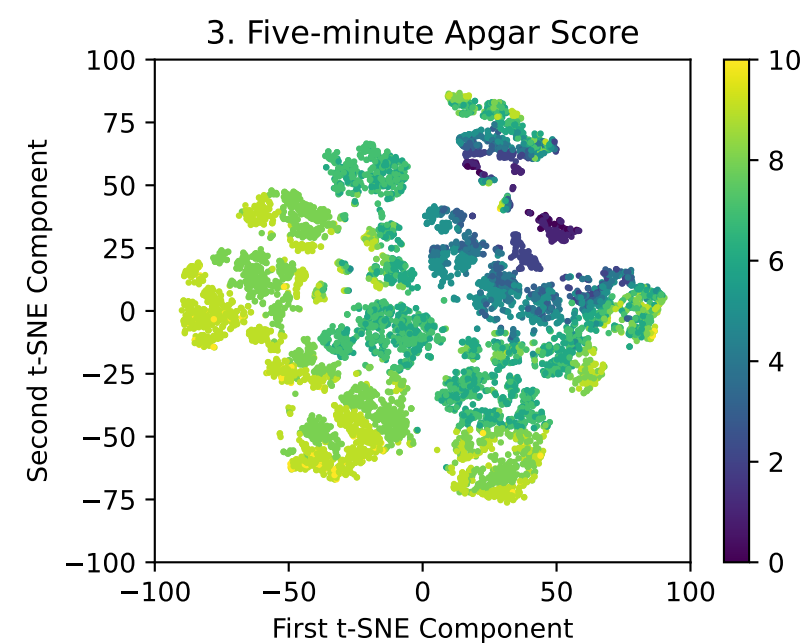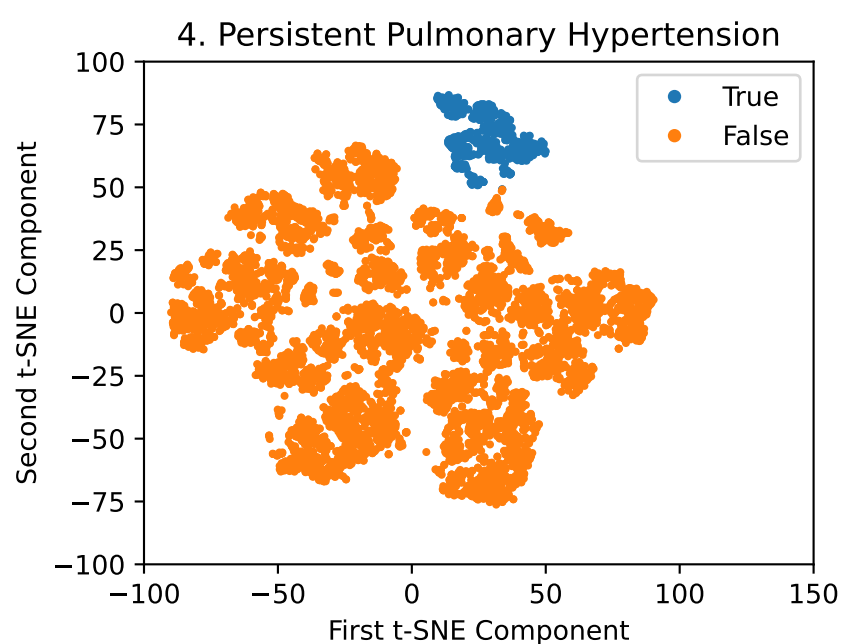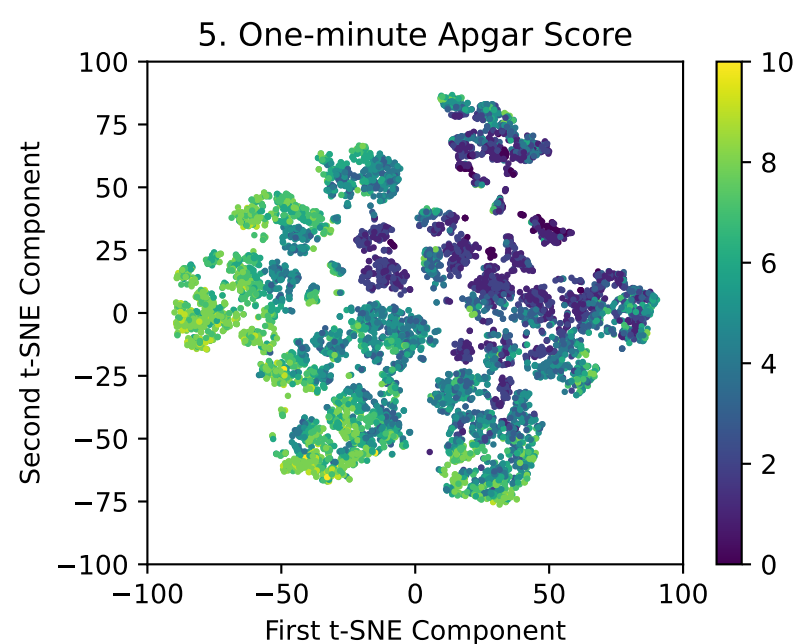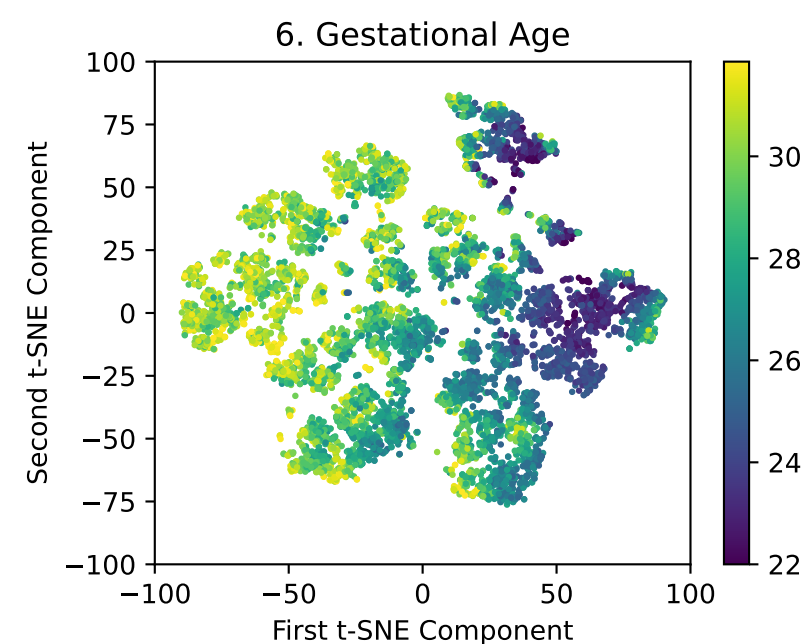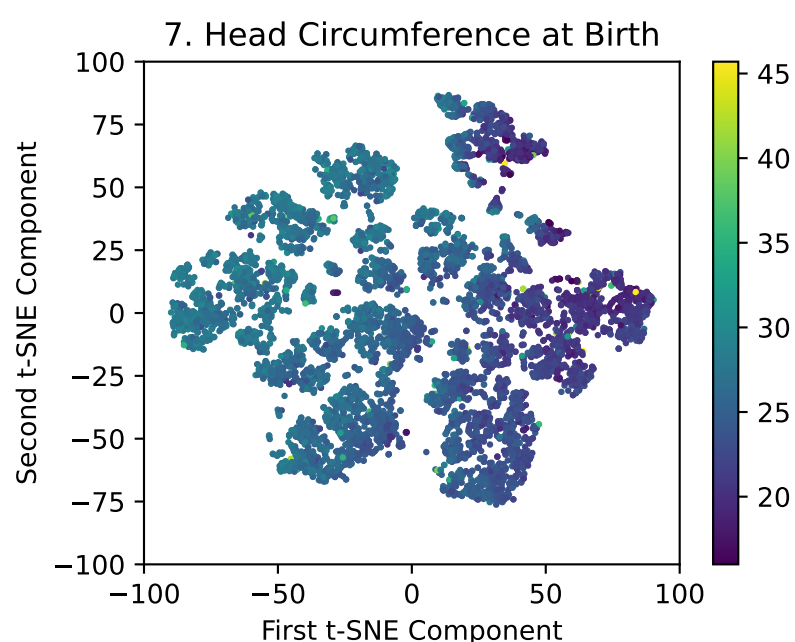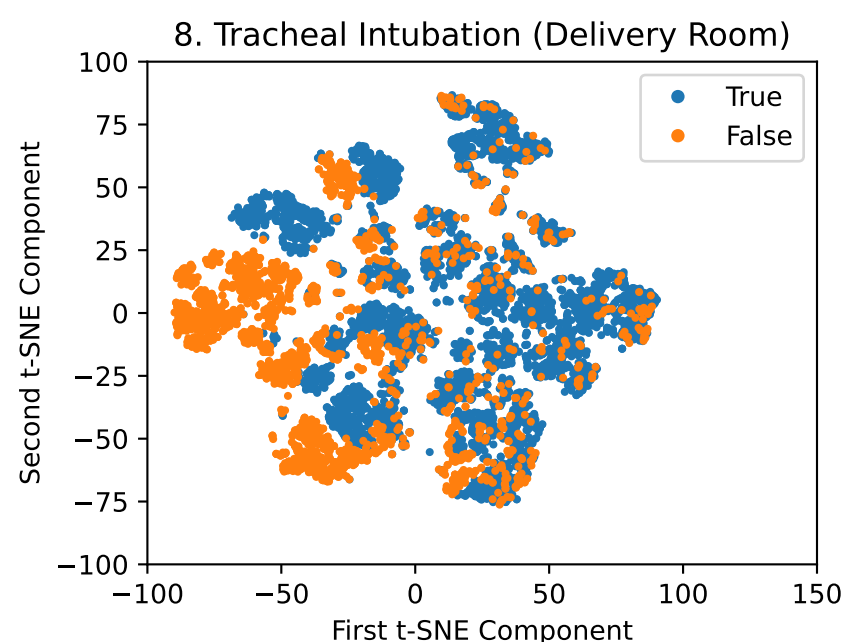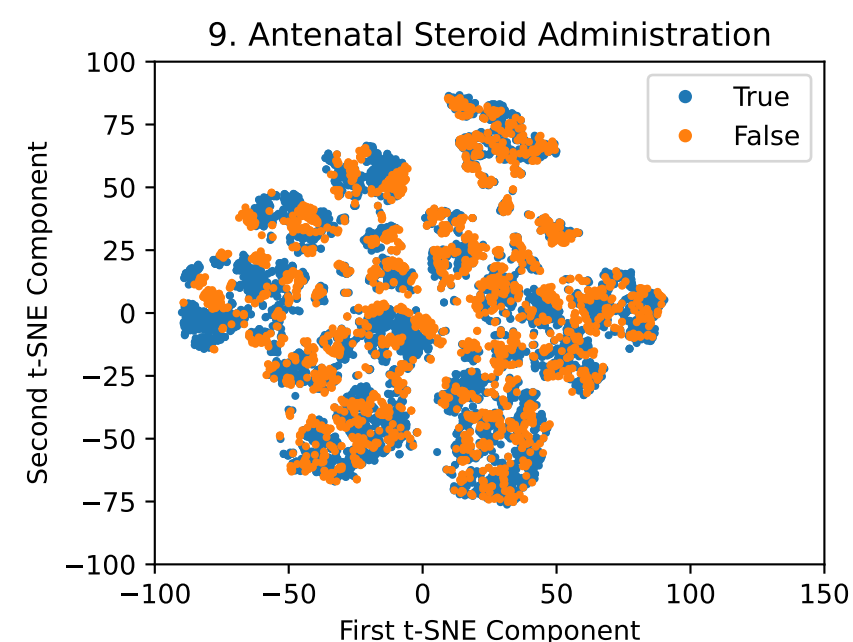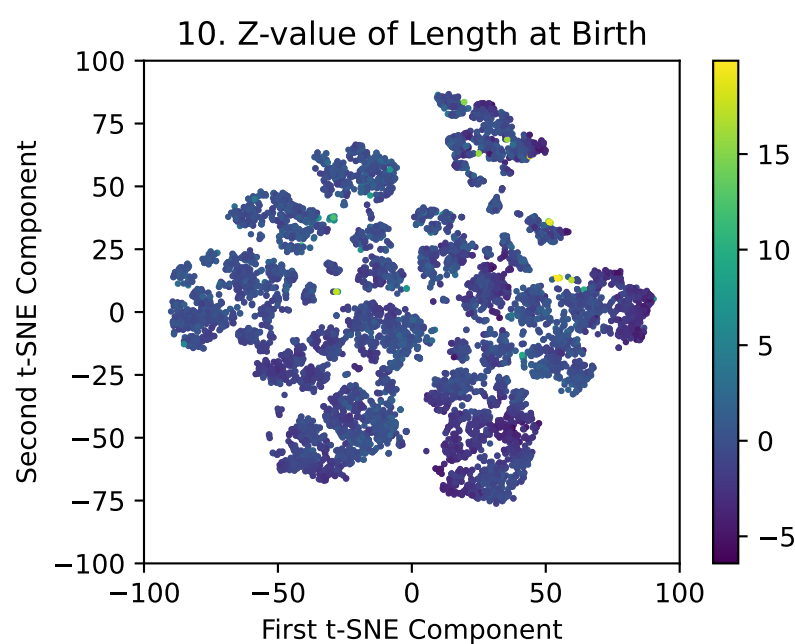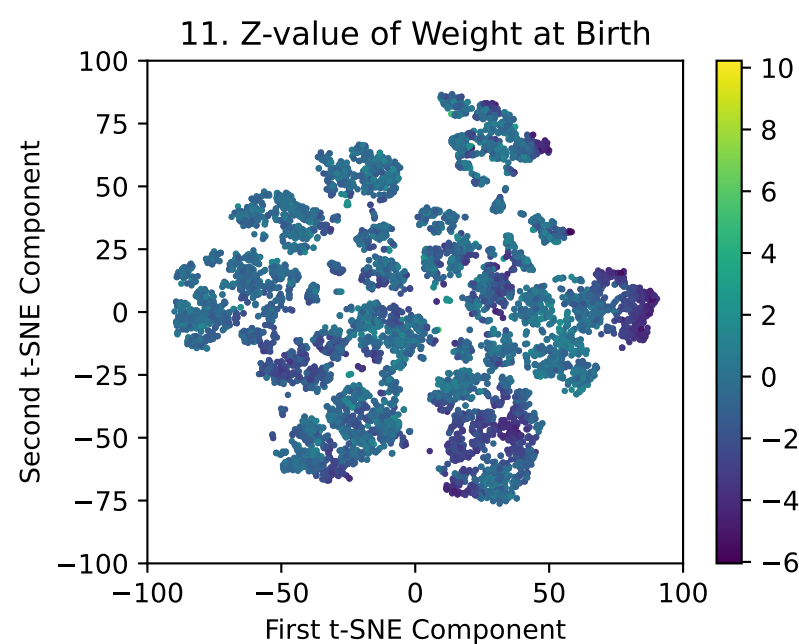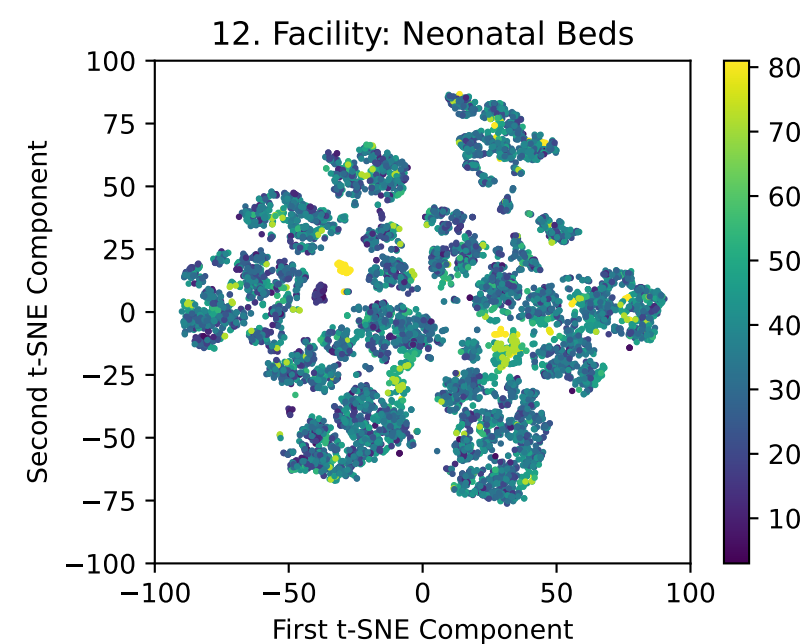

Supplement: S9 Fig — Derived from the SHAP values of the first imputed test set. (PDF) [file pone.0300817.s009.pdf]

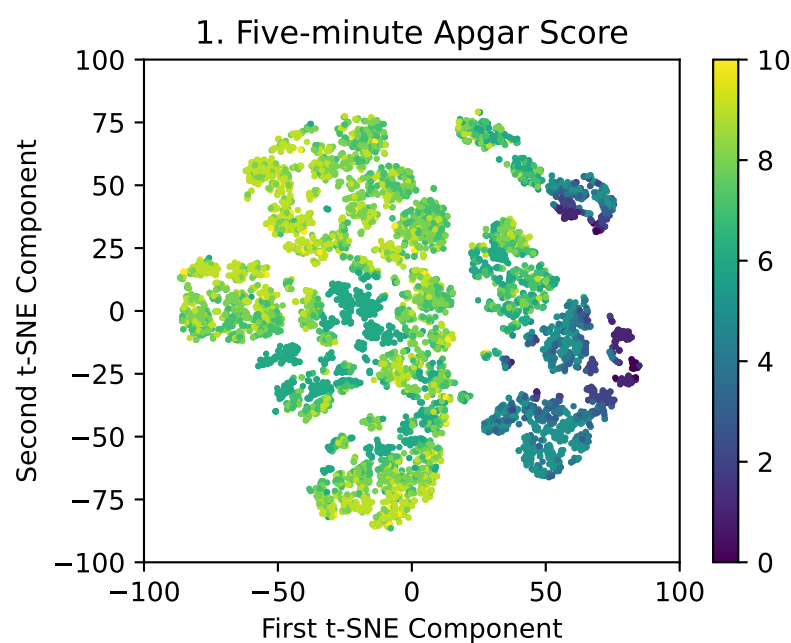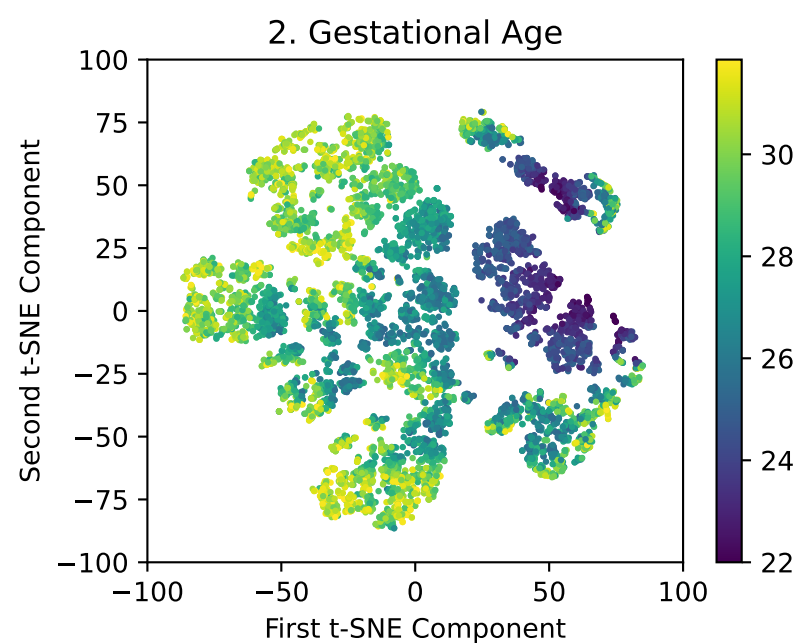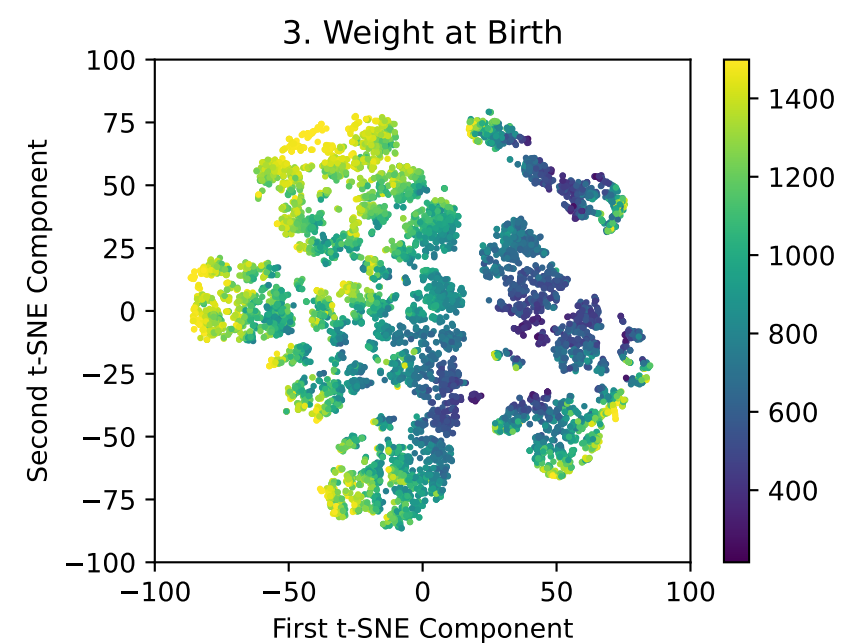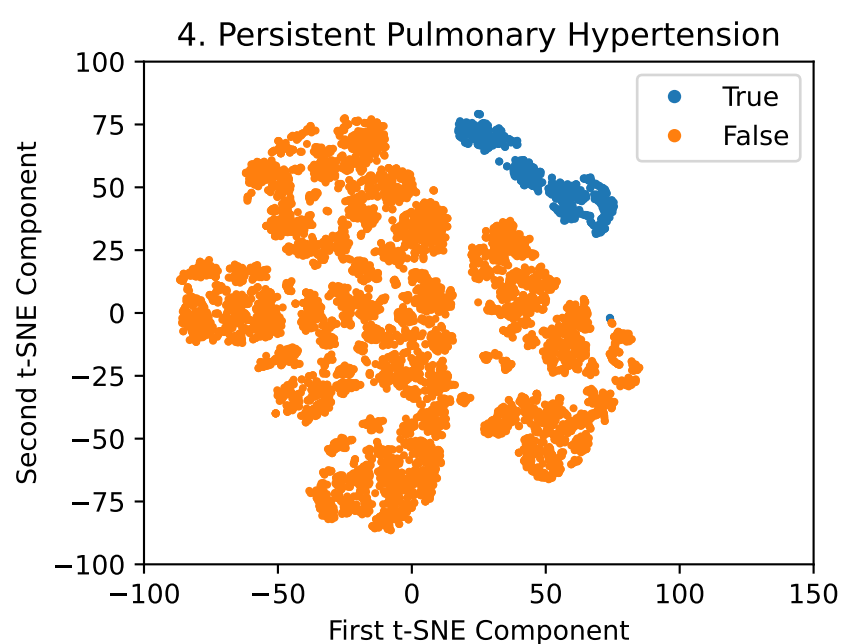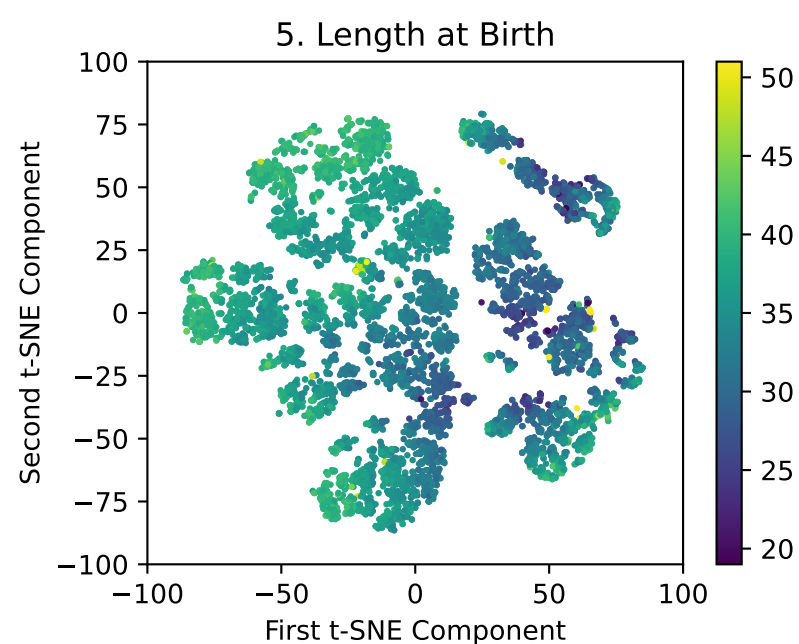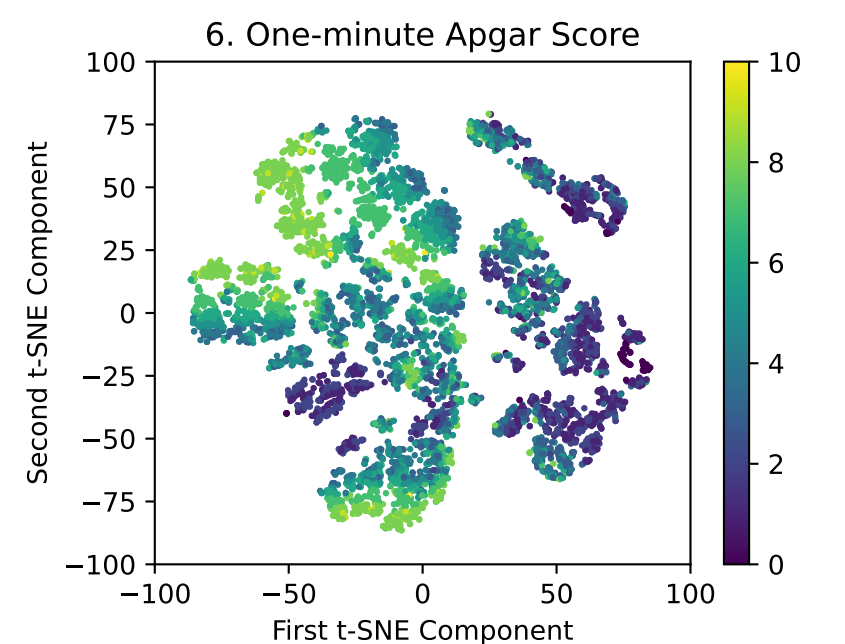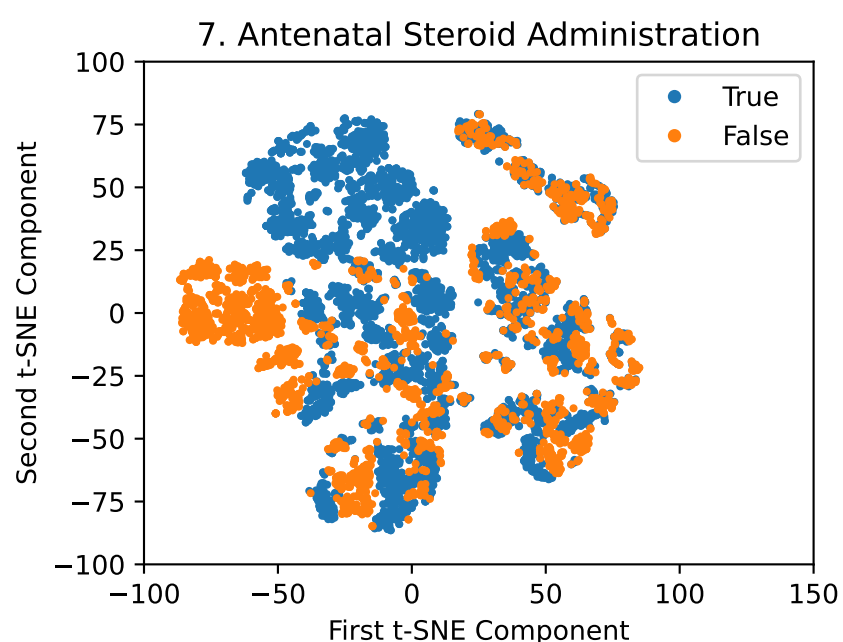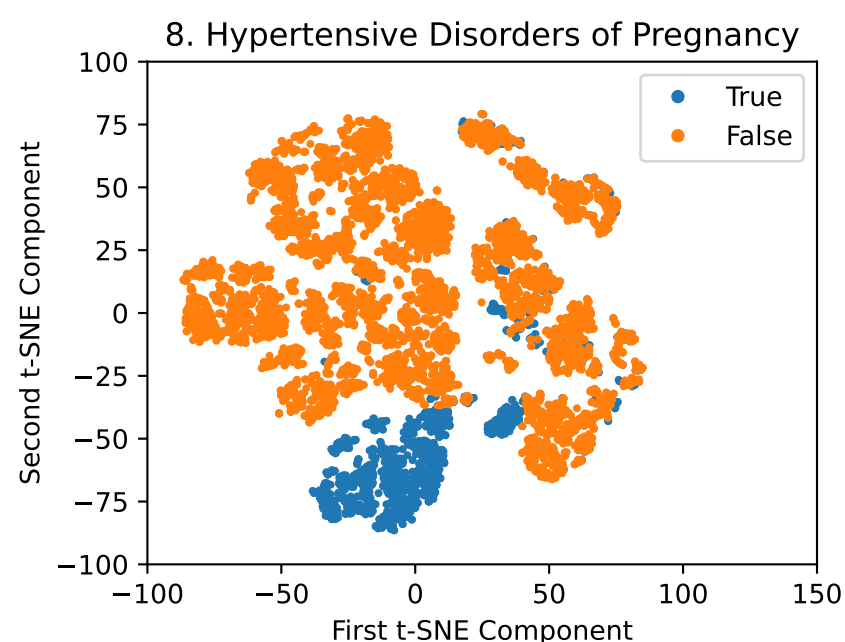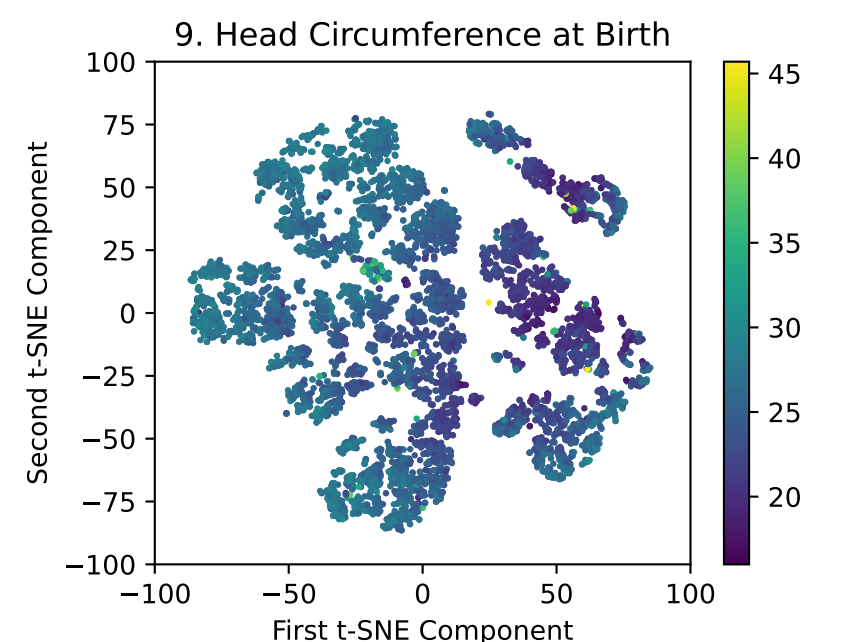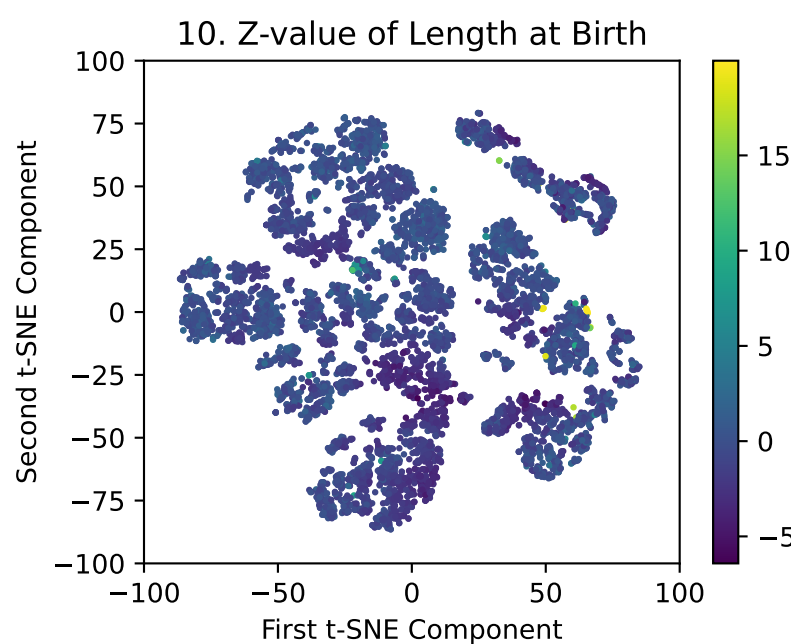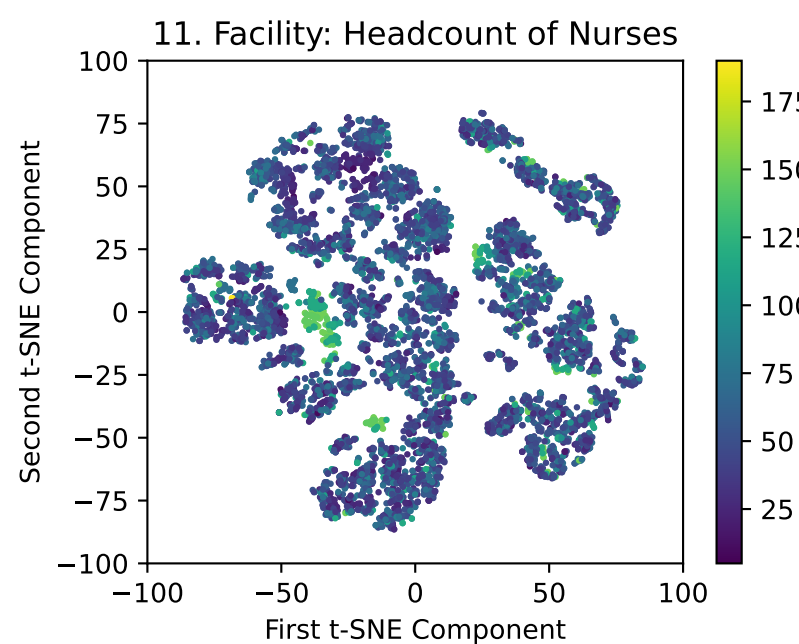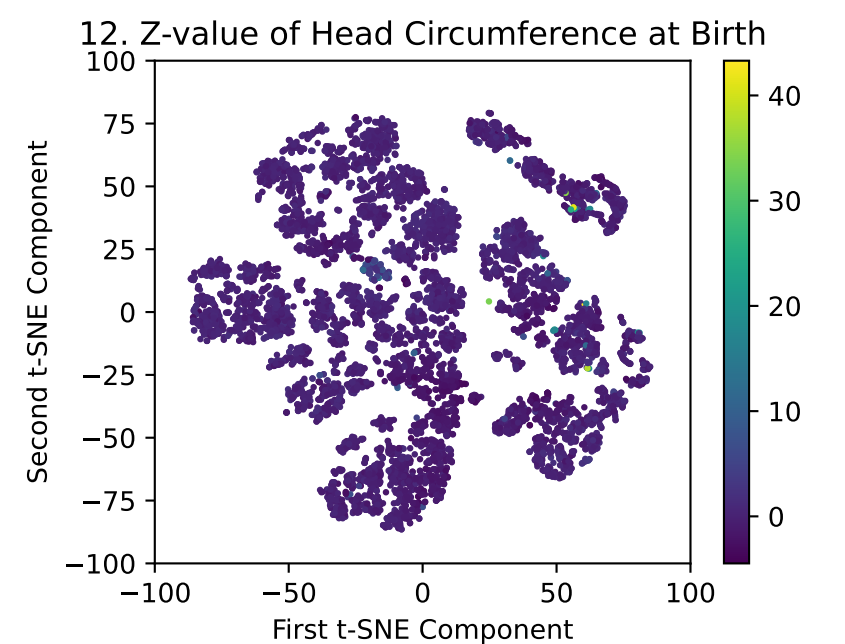

Supplement: S10 Fig — Derived from the SHAP values of the first imputed test set. (PDF) [file pone.0300817.s010.pdf]
