# Supplementary material for: Immediate postnatal prediction of death or bronchopulmonary dysplasia among very preterm and very low birth weight infants based on gradient boosting decision trees algorithm: A nationwide database study in Japan
Source: PLoS One. 2024 Mar 27;19(3):e0300817. doi: 10.1371/journal.pone.0300817 (PMC10971761; doi:10.1371/journal.pone.0300817)
Supplement: S5 Fig — Derived from the SHAP values of the 20 imputed test sets. (PDF) [file pone.0300817.s005.pdf]

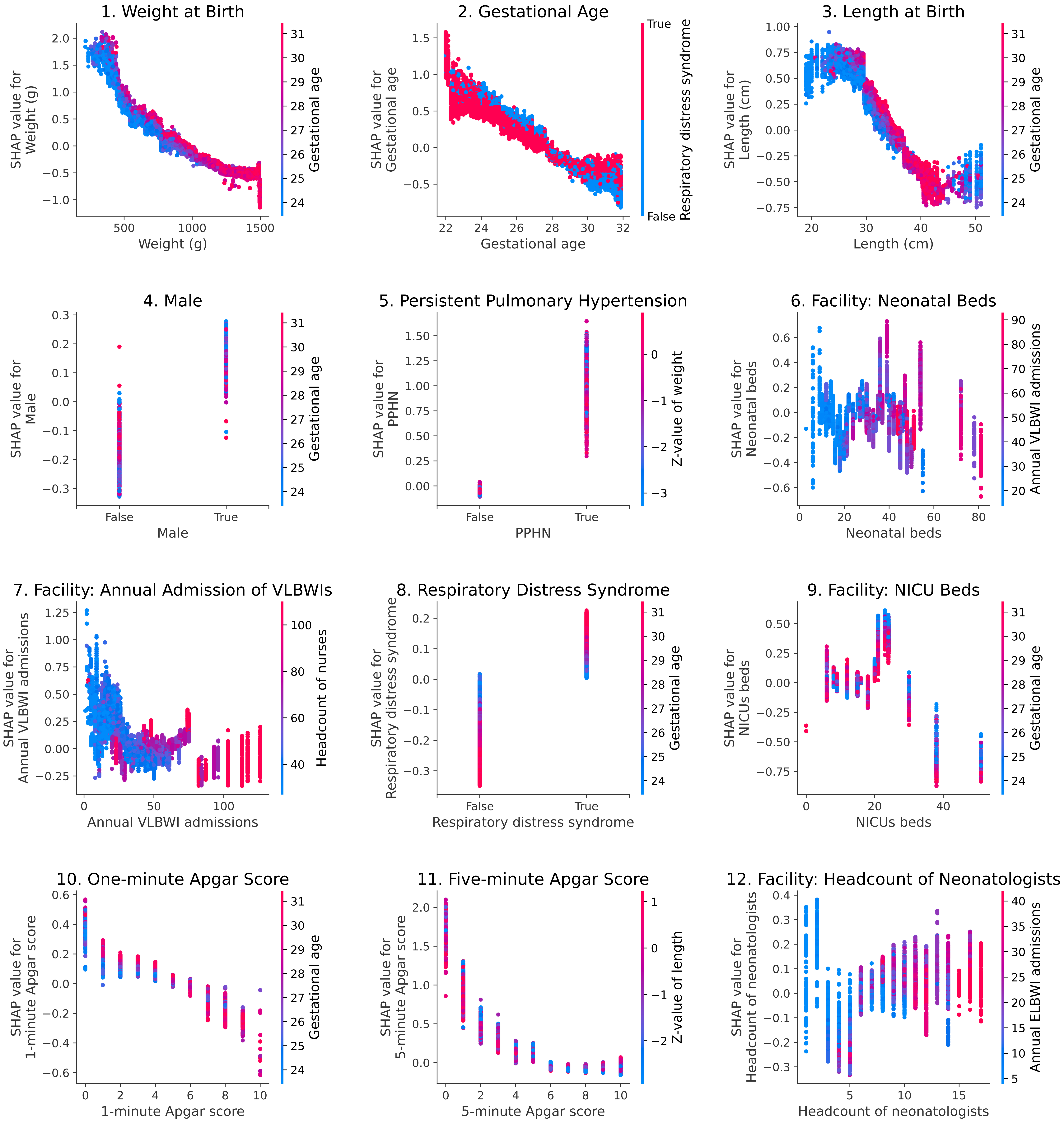
1. Weight at Birth
SHAP value for Weight (g)
Weight (g)
Gestational age
2. Gestational Age
SHAP value for Gestational age
Gestational age
Respiratory distress syndrome
True
False
3. Length at Birth
SHAP value for Length (cm)
Length (cm)
Gestational age
4. Male
SHAP value for Male
Male
Gestational age
5. Persistent Pulmonary Hypertension
SHAP value for PPHN
PPHN
Z-value of weight
6. Facility: Neonatal Beds
SHAP value for Neonatal beds
Neonatal beds
Annual VLBWI admissions
7. Facility: Annual Admission of VLBWIs
SHAP value for Annual VLBWI admissions
Annual VLBWI admissions
Headcount of nurses
8. Respiratory Distress Syndrome
SHAP value for Respiratory distress syndrome
Respiratory distress syndrome
Gestational age
9. Facility: NICU Beds
SHAP value for NICUs beds
NICUs beds
Gestational age
10. One-minute Apgar Score
SHAP value for 1-minute Apgar score
1-minute Apgar score
Gestational age
11. Five-minute Apgar Score
SHAP value for 5-minute Apgar score
5-minute Apgar score
Z-value of length
12. Facility: Headcount of Neonatologists
SHAP value for Headcount of neonatologists
Headcount of neonatologists
Annual ELBWI admissions
