# Supplementary material for: Immediate postnatal prediction of death or bronchopulmonary dysplasia among very preterm and very low birth weight infants based on gradient boosting decision trees algorithm: A nationwide database study in Japan
Source: PLoS One. 2024 Mar 27;19(3):e0300817. doi: 10.1371/journal.pone.0300817 (PMC10971761; doi:10.1371/journal.pone.0300817)
Supplement: S6 Fig — Derived from the SHAP values of the 20 imputed test sets. (PDF) [file pone.0300817.s006.pdf]

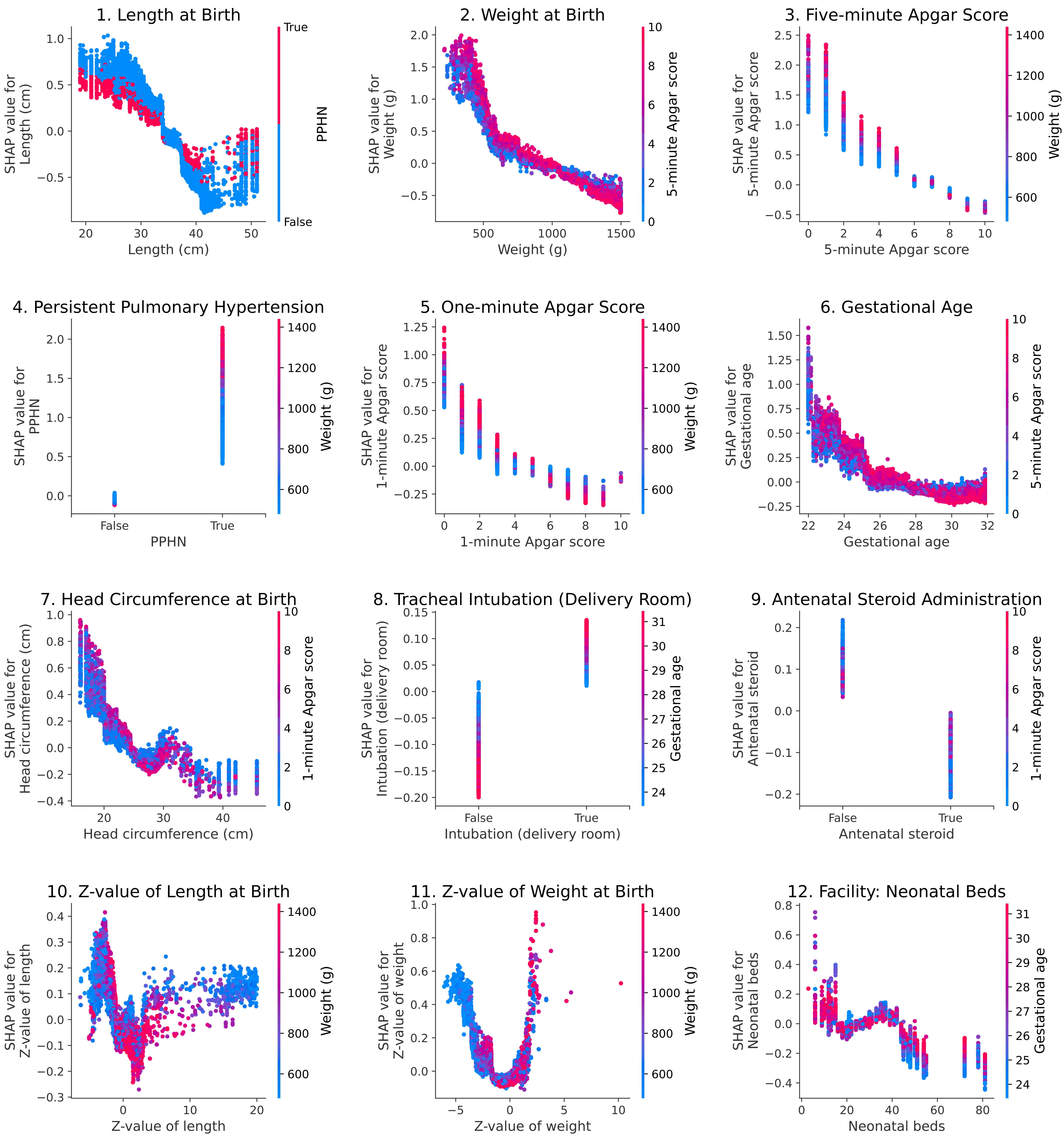
1. Length at Birth
SHAP value for Length (cm)
Length (cm)
PPHN
True
False
2. Weight at Birth
SHAP value for Weight (g)
Weight (g)
5-minute Apgar score
3. Five-minute Apgar Score
SHAP value for 5-minute Apgar score
5-minute Apgar score
Weight (g)
4. Persistent Pulmonary Hypertension
SHAP value for PPHN
PPHN
False
True
Weight (g)
5. One-minute Apgar Score
SHAP value for 1-minute Apgar score
1-minute Apgar score
Weight (g)
6. Gestational Age
SHAP value for Gestational age
Gestational age
5-minute Apgar score
7. Head Circumference at Birth
SHAP value for Head circumference (cm)
Head circumference (cm)
1-minute Apgar score
8. Tracheal Intubation (Delivery Room)
SHAP value for Intubation (delivery room)
Intubation (delivery room)
False
True
Gestational age
9. Antenatal Steroid Administration
SHAP value for Antenatal steroid
Antenatal steroid
False
True
1-minute Apgar score
10. Z-value of Length at Birth
SHAP value for Z-value of length
Z-value of length
Weight (g)
11. Z-value of Weight at Birth
SHAP value for Z-value of weight
Z-value of weight
Weight (g)
12. Facility: Neonatal Beds
SHAP value for Neonatal beds
Neonatal beds
Gestational age
