# Supplementary material for: Immediate postnatal prediction of death or bronchopulmonary dysplasia among very preterm and very low birth weight infants based on gradient boosting decision trees algorithm: A nationwide database study in Japan
Source: PLoS One. 2024 Mar 27;19(3):e0300817. doi: 10.1371/journal.pone.0300817 (PMC10971761; doi:10.1371/journal.pone.0300817)
Supplement: S7 Fig — Derived from the SHAP values of the 20 imputed test sets. (PDF) [file pone.0300817.s007.pdf]

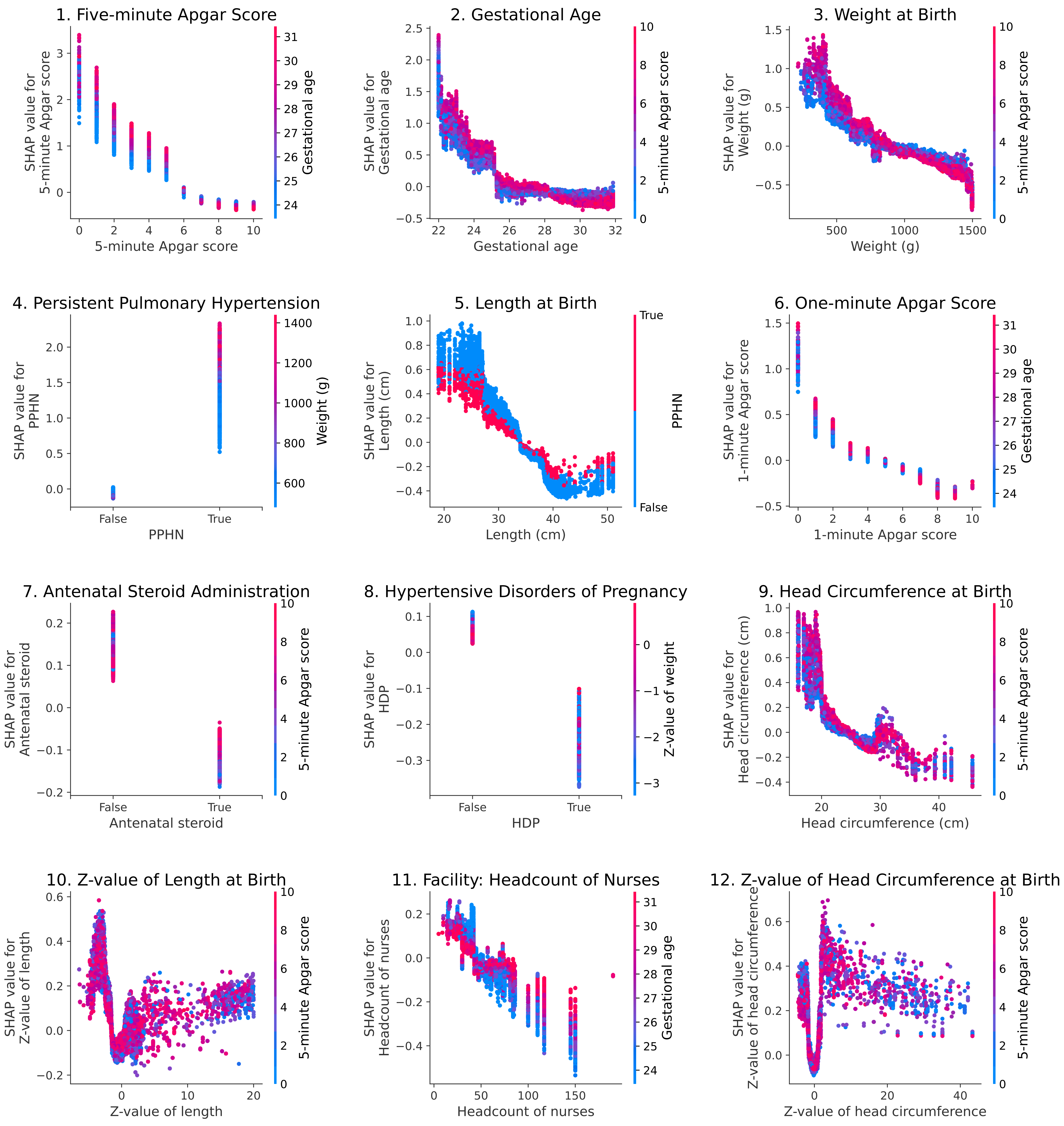
1. Five-minute Apgar Score
SHAP value for 5-minute Apgar score
5-minute Apgar score
Gestational age
2. Gestational Age
SHAP value for Gestational age
Gestational age
5-minute Apgar score
3. Weight at Birth
SHAP value for Weight (g)
Weight (g)
5-minute Apgar score
4. Persistent Pulmonary Hypertension
SHAP value for PPHN
PPHN
False
True
Weight (g)
5. Length at Birth
SHAP value for Length (cm)
Length (cm)
PPHN
True
False
6. One-minute Apgar Score
SHAP value for 1-minute Apgar score
1-minute Apgar score
Gestational age
7. Antenatal Steroid Administration
SHAP value for Antenatal steroid
Antenatal steroid
False
True
5-minute Apgar score
8. Hypertensive Disorders of Pregnancy
SHAP value for HDP
HDP
False
True
Z-value of weight
9. Head Circumference at Birth
SHAP value for Head circumference (cm)
Head circumference (cm)
5-minute Apgar score
10. Z-value of Length at Birth
SHAP value for Z-value of length
Z-value of length
5-minute Apgar score
11. Facility: Headcount of Nurses
SHAP value for Headcount of nurses
Headcount of nurses
Gestational age
12. Z-value of Head Circumference at Birth
SHAP value for Z-value of head circumference
Z-value of head circumference
5-minute Apgar score
