# Supplementary material for: Immediate postnatal prediction of death or bronchopulmonary dysplasia among very preterm and very low birth weight infants based on gradient boosting decision trees algorithm: A nationwide database study in Japan
Source: PLoS One. 2024 Mar 27;19(3):e0300817. doi: 10.1371/journal.pone.0300817 (PMC10971761; doi:10.1371/journal.pone.0300817)
Supplement: S1 Table — (DOCX) [file pone.0300817.s011.docx]

S1 Table. Univariable and Multivariable Logistic Regression for Death or Bronchopulmonary Dysplasia.

|  | **Univariable** | | | **Multivariable** | | | |
| --- | --- | --- | --- | --- | --- | --- | --- |
| **Characteristic** | **OR**^1^ | **95% CI**^1^ | **p-value** | **OR**^1^ | **95% CI**^1^ | **p-value** | **Adjusted GVIF**^12^ |
| **Maternal age** | 1.00 | 1.00, 1.00 | 0.9 | 1.00 | 1.00, 1.01 | 0.5 | 1.1 |
| **Gravidity** | 1.01 | 0.99, 1.02 | 0.4 | 0.99 | 0.96, 1.02 | 0.6 | 1.5 |
| **Parity** | 0.98 | 0.95, 1.00 | 0.094 | 0.97 | 0.92, 1.02 | 0.3 | 1.5 |
| **Number of fetuses** | 0.84 | 0.80, 0.88 | <0.001 | 0.91 | 0.81, 1.01 | 0.066 | 1.6 |
| **Birth order** | 0.90 | 0.84, 0.96 | 0.002 | 1.07 | 0.94, 1.21 | 0.3 | 1.4 |
| **Monochorionic multiple** | 0.98 | 0.90, 1.06 | 0.5 | 1.25 | 1.10, 1.41 | <0.001 | 1.2 |
| **Maternal diabetes** | 1.01 | 0.89, 1.15 | 0.9 | 1.10 | 0.94, 1.30 | 0.2 | 1.0 |
| **Hypertensive disorders of pregnancy** | 0.83 | 0.78, 0.88 | <0.001 | 0.90 | 0.82, 0.99 | 0.025 | 1.2 |
| **Clinical chorioamnionitis** | 1.52 | 1.43, 1.61 | <0.001 | 1.09 | 1.01, 1.19 | 0.034 | 1.1 |
| **Premature rupture of membranes** | 1.15 | 1.09, 1.21 | <0.001 | 1.06 | 0.98, 1.14 | 0.2 | 1.1 |
| **Antenatal steroid administration** | 1.08 | 1.03, 1.14 | 0.001 | 1.17 | 1.09, 1.25 | <0.001 | 1.0 |
| **Non-reassuring fetal status** | 1.30 | 1.23, 1.37 | <0.001 | 1.03 | 0.95, 1.11 | 0.5 | 1.1 |
| **Cephalic presentation** | 0.73 | 0.70, 0.77 | <0.001 | 0.96 | 0.90, 1.03 | 0.2 | 1.1 |
| **Delivery mode** |  |  |  |  |  |  | 1.1 |
| Caesarean section | — | — |  | — | — |  |  |
| Natural vaginal delivery | 1.02 | 0.96, 1.08 | 0.5 | 0.94 | 0.86, 1.02 | 0.15 |  |
| Forceps or vacuum delivery | 1.19 | 0.86, 1.63 | 0.3 | 1.02 | 0.63, 1.60 | >0.9 |  |
| **Transport pathway** |  |  |  |  |  |  | 1.1 |
| Maternal inpatient transport | — | — |  | — | — |  |  |
| Maternal outpatient referral | 0.91 | 0.86, 0.97 | 0.001 | 1.12 | 1.03, 1.21 | 0.006 |  |
| Neonatal transport | 0.93 | 0.83, 1.04 | 0.2 | 1.15 | 0.96, 1.36 | 0.12 |  |
| Without referral | 0.92 | 0.85, 0.99 | 0.027 | 1.08 | 0.97, 1.20 | 0.2 |  |
| **Gestational age** | 0.67 | 0.66, 0.68 | <0.001 | 0.94 | 0.83, 1.06 | 0.3 | 9.0 |
| **Male sex** | 1.20 | 1.15, 1.26 | <0.001 | 1.27 | 1.19, 1.37 | <0.001 | 1.2 |
| **Apgar score at 1 min.** | 0.77 | 0.77, 0.78 | <0.001 | 0.96 | 0.94, 0.98 | <0.001 | 1.6 |
| **Apgar score at 5 min.** | 0.75 | 0.74, 0.76 | <0.001 | 0.97 | 0.95, 0.99 | 0.010 | 1.5 |
| **Oxygen administration in the delivery room** | 1.53 | 1.42, 1.65 | <0.001 | 1.15 | 1.01, 1.30 | 0.031 | 1.0 |
| **Intubation in the delivery room** | 3.32 | 3.14, 3.52 | <0.001 | 1.16 | 1.07, 1.27 | <0.001 | 1.2 |
| **Cord blood transfusion** | 1.59 | 1.51, 1.68 | <0.001 | 1.01 | 0.94, 1.09 | 0.7 | 1.1 |
| **Weight at birth** | 1.00 | 1.00, 1.00 | <0.001 | 1.00 | 1.00, 1.00 | 0.6 | 6.4 |
| **Length at birth** | 0.77 | 0.76, 0.78 | <0.001 | 0.82 | 0.76, 0.89 | <0.001 | 8.8 |
| **Head circumference at birth** | 0.69 | 0.68, 0.70 | <0.001 | 0.94 | 0.86, 1.03 | 0.2 | 7.3 |
| **Z-value of weight at birth** | 0.90 | 0.88, 0.91 | <0.001 | 0.80 | 0.72, 0.89 | <0.001 | 4.6 |
| **Z-value of length at birth** | 0.92 | 0.90, 0.94 | <0.001 | 1.30 | 1.12, 1.52 | <0.001 | 6.7 |
| **Z-value of head circumference at birth** | 0.92 | 0.90, 0.94 | <0.001 | 1.06 | 0.94, 1.20 | 0.4 | 4.7 |
| **Respiratory distress syndrome** | 2.05 | 1.94, 2.17 | <0.001 | 1.15 | 1.07, 1.25 | <0.001 | 1.1 |
| **Persistent pulmonary hypertension of the newborn** | 4.48 | 4.06, 4.95 | <0.001 | 2.20 | 1.94, 2.51 | <0.001 | 1.0 |
| **Hypoxic-ischemic encephalopathy** | 3.46 | 2.75, 4.38 | <0.001 | 2.34 | 1.69, 3.24 | <0.001 | 1.0 |
| **Facility level** |  |  |  |  |  |  | 1.3 |
| Tertiary | — | — |  | — | — |  |  |
| Secondary | 0.94 | 0.88, 0.99 | 0.026 | 1.25 | 1.12, 1.40 | <0.001 |  |
| Primary | 0.96 | 0.81, 1.14 | 0.6 | 1.04 | 0.79, 1.35 | 0.8 |  |
| **Facility provider** |  |  |  |  |  |  | 1.1 |
| Public hospital | — | — |  | — | — |  |  |
| Private hospital | 1.17 | 1.10, 1.24 | <0.001 | 1.27 | 1.17, 1.38 | <0.001 |  |
| Independent administrative agency | 1.04 | 0.97, 1.11 | 0.3 | 1.05 | 0.96, 1.14 | 0.3 |  |
| National hospital | 0.85 | 0.76, 0.96 | 0.007 | 0.81 | 0.69, 0.94 | 0.006 |  |
| **Annual admission of very low birth weight infants** | 1.00 | 1.00, 1.00 | >0.9 | 0.99 | 0.99, 1.00 | 0.003 | 3.1 |
| **Annual admission of extremely low birth weight infants** | 1.00 | 1.00, 1.01 | <0.001 | 1.00 | 1.00, 1.01 | 0.5 | 2.9 |
| **Number of beds for neonates** | 1.00 | 1.00, 1.01 | <0.001 | 1.00 | 1.00, 1.01 | 0.7 | 1.8 |
| **Number of beds in neonatal intensive care unit** | 1.01 | 1.00, 1.01 | 0.002 | 0.99 | 0.98, 1.00 | 0.029 | 2.0 |
| **Number of beds in maternal-fetal intensive care unit** | 1.01 | 1.00, 1.01 | 0.004 | 1.00 | 0.99, 1.01 | 0.7 | 1.4 |
| **Headcount of neonatologists** | 1.02 | 1.01, 1.03 | <0.001 | 1.00 | 0.99, 1.02 | >0.9 | 1.6 |
| **Headcount of nurses** | 1.00 | 1.00, 1.00 | <0.001 | 1.01 | 1.00, 1.01 | <0.001 | 2.2 |
| **Availability of psychologists** | 1.07 | 1.01, 1.13 | 0.012 | 1.00 | 0.92, 1.08 | >0.9 | 1.1 |
| **Availability of pediatric surgery** | 1.11 | 1.05, 1.18 | <0.001 | 0.97 | 0.87, 1.07 | 0.5 | 1.2 |
| **Availability of cardiac surgery** | 1.05 | 1.00, 1.10 | 0.030 | 1.00 | 0.93, 1.08 | >0.9 | 1.2 |
| **Availability of neurosurgery** | 1.11 | 1.05, 1.18 | <0.001 | 1.00 | 0.91, 1.09 | >0.9 | 1.2 |
| **Availability of ophthalmologists** | 1.68 | 1.44, 1.96 | <0.001 | 1.41 | 1.14, 1.75 | 0.002 | 1.0 |
| **Availability of a follow-up system** | 1.07 | 1.00, 1.13 | 0.037 | 0.86 | 0.79, 0.94 | <0.001 | 1.1 |
| ^1^OR = Odds Ratio, CI = Confidence Interval, GVIF = Generalized Variance Inflation Factor | | | | | | | |
| ^2^GVIF^[1/(2*df)] | | | | | | | |
