# Supplementary material for: Immediate postnatal prediction of death or bronchopulmonary dysplasia among very preterm and very low birth weight infants based on gradient boosting decision trees algorithm: A nationwide database study in Japan
Source: PLoS One. 2024 Mar 27;19(3):e0300817. doi: 10.1371/journal.pone.0300817 (PMC10971761; doi:10.1371/journal.pone.0300817)
Supplement: S2 Table — (DOCX) [file pone.0300817.s012.docx]

S2 Table. Characteristics of Clusters Based on Prediction for Death or Bronchopulmonary Dysplasia.

| **Characteristic** | **Cluster 1,^1^  N = 3,634** | **Cluster 2,^1^  N = 2,818** | **Cluster 3,^1^ N = 669** | **Cluster 4,^1^ N = 1,830** |
| --- | --- | --- | --- | --- |
| **Death or bronchopulmonary dysplasia** | 505 (14%) | 1,156 (41%) | 472 (71%) | 1,385 (76%) |
| **Death before discharge** | 45 (1.2%) | 90 (3.2%) | 158 (24%) | 300 (16%) |
| **Gestational age** | 30.00 (29.14, 30.86) | 27.29 (26.29, 28.43) | 25.86 (24.14, 28.14) | 24.43 (23.57, 25.71) |
| **Weight at birth** | 1,267 (1,148, 1,380) | 869 (788, 960) | 743 (594, 1,016) | 568 (489, 639) |
| **1-minute  Apgar score** | 6 (4, 8) | 4 (3, 6) | 3 (1, 4) | 3 (2, 5) |
| **5-minute  Apgar score** | 8 (7, 9) | 7 (6, 8) | 6 (4, 7) | 6 (4, 7) |
| **Persistent pulmonary hypertension** | 2 (<0.1%) | 0 (0%) | 669 (100%) | 93 (5.1%) |
| ^1^n (%); Median (IQR) | | | | |
