# Supplementary material for: Immediate postnatal prediction of death or bronchopulmonary dysplasia among very preterm and very low birth weight infants based on gradient boosting decision trees algorithm: A nationwide database study in Japan
Source: PLoS One. 2024 Mar 27;19(3):e0300817. doi: 10.1371/journal.pone.0300817 (PMC10971761; doi:10.1371/journal.pone.0300817)
Supplement: S3 Table — (DOCX) [file pone.0300817.s013.docx]

S3 Table. Characteristics of Clusters Based on Prediction for Death or Severe Bronchopulmonary Dysplasia.

| **Characteristic** | **Cluster 1,^1^ N = 4,466** | **Cluster 2,^1^ N = 2,790** | **Cluster 3,^1^ N = 880** | **Cluster 4,^1^ N = 698** |
| --- | --- | --- | --- | --- |
| **Death or severe bronchopulmonary dysplasia** | 60 (1.3%) | 386 (14%) | 175 (20%) | 216 (31%) |
| **Death before discharge** | 30 (0.7%) | 235 (8.4%) | 147 (17%) | 181 (26%) |
| **Gestational age** | 29.64 (28.43, 30.71) | 25.71 (24.43, 27.14) | 27.29 (25.57, 29.00) | 25.43 (23.89, 28.00) |
| **Weight at birth** | 1,190 (1,034, 1,342) | 678 (556, 788) | 887 (703, 1,104) | 705 (556, 980) |
| **1-minute  Apgar score** | 6 (4, 8) | 4 (2, 5) | 1 (1, 2) | 3 (1, 4) |
| **5-minute  Apgar score** | 8 (7, 9) | 7 (6, 8) | 3 (2, 5) | 6 (4, 7) |
| **Persistent pulmonary hypertension** | 0 (0%) | 15 (0.5%) | 23 (2.6%) | 698 (100%) |
| ^1^n (%); Median (IQR) | | | | |
