# Supplementary material for: Immediate postnatal prediction of death or bronchopulmonary dysplasia among very preterm and very low birth weight infants based on gradient boosting decision trees algorithm: A nationwide database study in Japan
Source: PLoS One. 2024 Mar 27;19(3):e0300817. doi: 10.1371/journal.pone.0300817 (PMC10971761; doi:10.1371/journal.pone.0300817)
Supplement: S4 Table — (DOCX) [file pone.0300817.s014.docx]

S4. Table. Characteristics of Clusters Based on Prediction for Death before Discharge.

| **Characteristic** | **Cluster 1,^1^ N = 5,518** | **Cluster 2,^1^ N = 1,792** | **Cluster 3,^1^ N = 1,205^1^** | **Cluster 4,^1^ N = 742^1^** |
| --- | --- | --- | --- | --- |
| **Death before discharge** | 43 (0.8%) | 189 (11%) | 178 (15%) | 183 (25%) |
| **Gestational age** | 29.29 (28.00, 30.57) | 24.86 (23.86, 26.00) | 27.00 (25.29, 28.86) | 25.57 (24.00, 28.14) |
| **Weight at birth** | 1,127 (939, 1,311) | 600 (517, 692) | 847 (667, 1,086) | 711 (556, 994) |
| **1-minute  Apgar score** | 6 (4, 7) | 4 (2, 5) | 2 (1, 3) | 3 (1, 4) |
| **5-minute  Apgar score** | 8 (7, 9) | 7 (6, 8) | 4 (2, 5) | 6 (4, 7) |
| **Persistent pulmonary hypertension** | 0 (0%) | 6 (0.3%) | 42 (3.5%) | 742 (100%) |
| ^1^n (%); Median (IQR) | | | | |
